# Association genetics studies on frost tolerance in wheat (*Triticum aestivum* L.) reveal new highly conserved amino acid substitutions in *CBF-A3*, *CBF-A15*, *VRN3* and *PPD1* genes

**DOI:** 10.1186/s12864-018-4795-6

**Published:** 2018-05-29

**Authors:** Steve Babben, Edgar Schliephake, Philipp Janitza, Thomas Berner, Jens Keilwagen, Michael Koch, Fernando Alberto Arana-Ceballos, Sven Eduard Templer, Yuriy Chesnokov, Tatyana Pshenichnikova, Jörg Schondelmaier, Andreas Börner, Klaus Pillen, Frank Ordon, Dragan Perovic

**Affiliations:** 1 Julius Kühn-Institut (JKI), Federal Research Centre for Cultivated Plants, Institute for Resistance Research and Stress Tolerance, Erwin-Baur-Str. 27, 06484 Quedlinburg, Saxony-Anhalt Germany; 2 Martin Luther University Halle-Wittenberg (MLU), Institute of Agricultural and Nutritional Sciences, Betty-Heimann-Str. 5, 06120 Halle (Saale), Saxony-Anhalt Germany; 3 Julius Kühn-Institut (JKI), Federal Research Centre for Cultivated Plants, Institute for Biosafety in Plant Biotechnology, Erwin-Baur-Str. 27, 06484 Quedlinburg, Saxony-Anhalt Germany; 4 Deutsche Saatveredelung AG (DSV), Weißenburger Str. 5, 59557 Lippstadt, Nordrhein-Westfalen Germany; 5 Leibniz Institute of Plant Genetics and Crop Plant Research (IPK), Resources Genetics and Reproduction, Correnstraße 3, 06466 Seeland OT Gatersleben, Saxony-Anhalt Germany; 6 Max Planck Institute for Biology of Ageing, Joseph-Stelzmann-Str. 9B, 50931 Cologne, Nordrhein-Westfalen Germany; 7 Agrophysical Research Institute (AFI), Grazhdanskii prosp. 14, 195220 St. Petersburg, Russia; 8 Institute of Cytology and Genetics of Siberian Branch of the Russian Academy of Sciences, Prospekt Lavrentyeva 10, 630090 Novosibirsk, Russia; 9 Saaten-Union Biotec GmbH, Hovedisser Str. 94, 33818 Leopoldshoehe, Nordrhein-Westfalen Germany; 10 Martin Luther University Halle-Wittenberg (MLU), Institute of Agricultural and Nutritional Sciences, Betty-Heimann-Str. 3, 06120 Halle (Saale), Saxony-Anhalt Germany

**Keywords:** *Triticum aestivum* L., Frost tolerance (FT), Candidate genes, Association studies, SNP, Indel, Haplotypes, *CBF*, *VRN*, *PPD1*

## Abstract

**Background:**

Understanding the genetic basis of frost tolerance (FT) in wheat (*Triticum aestivum* L.) is essential for preventing yield losses caused by frost due to cellular damage, dehydration and reduced metabolism. FT is a complex trait regulated by a number of genes and several gene families. Availability of the wheat genomic sequence opens new opportunities for exploring candidate genes diversity for FT. Therefore, the objectives of this study were to identity SNPs and insertion-deletion (indels) in genes known to be involved in frost tolerance and to perform association genetics analysis of respective SNPs and indels on FT.

**Results:**

Here we report on the sequence analysis of 19 candidate genes for FT in wheat assembled using the Chinese Spring IWGSC RefSeq v1.0. Out of these, the tandem duplicated C-repeat binding factors (*CBF*), i.e. *CBF-A3*, *CBF-A5*, *CBF-A10*, *CBF-A13*, *CBF-A14*, *CBF-A15*, *CBF-A18*, the vernalisation response gene *VRN-A1*, *VRN-B3*, the photoperiod response genes *PPD-B1* and *PPD-D1* revealed association to FT in 235 wheat cultivars. Within six genes (*CBF-A3*, *CBF-A15*, *VRN-A1*, *VRN-B3*, *PPD-B1* and *PPD-D1*) amino acid (AA) substitutions in important protein domains were identified. The amino acid substitution effect in *VRN-A1* on FT was confirmed and new AA substitutions in *CBF-A3*, *CBF-A15*, *VRN-B3*, *PPD-B1* and *PPD-D1* located at highly conserved sites were detected. Since these results rely on phenotypic data obtained at five locations in 2 years, detection of significant associations of FT to AA changes in *CBF-A3*, *CBF-A15*, *VRN-A1*, *VRN-B3*, *PPD-B1* and *PPD-D1* may be exploited in marker assisted breeding for frost tolerance in winter wheat.

**Conclusions:**

A set of 65 primer pairs for the genes mentioned above from a previous study was BLASTed against the IWGSC RefSeq resulting in the identification of 39 primer combinations covering the full length of 19 genes. This work demonstrates the usefulness of the IWGSC RefSeq in specific primer development for highly conserved gene families in hexaploid wheat and, that a candidate gene association genetics approach based on the sequence data is an efficient tool to identify new alleles of genes important for the response to abiotic stress in wheat.

**Electronic supplementary material:**

The online version of this article (10.1186/s12864-018-4795-6) contains supplementary material, which is available to authorized users.

## Background

Wheat (*Triticum aestivum* L.) is on the world wide level the crop grown on the largest acreage and is of prime importance for human nutrition and animal feed. Worldwide production of 729 million tones in 2014, ranks wheat globally the third most important crop, next to maize (*Zea mays* L.) and rice (*Oryza sativa* L.). Production of wheat in Europe was 249.13 mt with an average yield of 4.25 t/ha in 2014. At the same time in North America 84.68 mt of wheat were harvested with an average yield of 2.994 t/ha, while in Asia the average yield was 3.095 t/ha resulting in a production of 315.71 mt [1]. In North America, North and Eastern Europe and Russia wheat production is exposed to low temperature and frost damage occurs frequently. Depending of the time of sowing wheat is known as winter or spring wheat, respectively, which differ in the vernalisation requirement and frost hardiness. Winter wheat varieties require an extended exposure to cold temperatures, typically 4 to 8 weeks at less than 4 °C, to induce flowering (vernalization) while spring wheat varieties do not have such a requirement [2]. Vernalization requirements keep these plants safely dormant over winter resulting in a longer vegetation period and higher yields of winter wheat.

### Wheat genome

Bread wheat is an allohexaploid species (2n = 6× = 42) with an AABBDD genome derived from two independent hybridisations [3–5]. This complex genome of about 17 Giga-base pairs (Gbp) has a repeat content of approximately 80% [6] with a gene density of on average one gene per 87 to 184 Kilo-base pairs (Kbp) [7]. The acquisition of this large genome sequence became feasible since the introduction of next Generation Sequencing (NGS) technologies.

Nowadays, the efforts of the International Wheat Genome Sequencing Consortium (IWGSC), in the development of a physical map and a reference sequence facilitates many downstream applications, i.e. development of high throughput genotyping platforms [8], efficient development of genome specific primers [9], exome sequencing in large genebank collections [10] etc. The wheat reference sequence, survey sequence and physical map are available at many public data bases. The CerealsDB web page, created by members of the Functional Genomics Group at the University of Bristol [11], includes online resources of genomic information, i.e. varietal SNPs, DArT markers, and EST sequences all linked to a draft genome sequence of the cultivar Chinese Spring [12]. The Unité de Recherche Génomique Info (URGI) web portal includes datasets such as chromosome survey sequences, reference sequences, physical maps, genetic maps, polymorphisms, genetic resources, extensive phenotypic data and various genomic arrays [13]. The chromosomal sequence information is available from the IWGSC [14]. All mentioned databases are useful for the identification of homologous chromosome sequences in bread wheat. Summarising, a lot of sequence information of wheat sorted chromosome arms [15–17], *T. urartu* [18] and *Ae. tauschii* [19] have been published during the past few years and are integrated in the public databases mentioned above. Today, the current version of the Chinese Spring IWGSC RefSeq v1.0 [14] facilitates the exploitation of the wheat sequence in basic science as well as in applied wheat breeding.

### Association analysis

Genome-wide association studies (GWAS) are a powerful tool to identify genomic regions and candidate genes involved in FT. High density genotyping arrays i.e. the Illumina 90 K chip [8], the Affymetrix 820 K [20] and the breeding 35 K axiom [21] arrays enable the determination of the genetic structure of complex traits by GWAS. However, they are limited in the identification of new alleles involved in FT. One approach that allows mining of novel alleles is re-sequencing of candidate genes followed by a candidate gene association genetics study [22].

### Cold stress signalling and transcriptional regulation

Frost tolerance (FT) is a complex biological process involving at least two main pathways and many additional processes encompassing a large number of genes. The main pathway is frost response, whereas flowering as the second pathway involves vernalisation. Plant cells can perceive cold stress through the membrane rigidification effect. This leads to Ca^2+^ influx into the cytosol and these characteristic Ca^2+^ signatures are detected by calcium binding proteins (CBPs) [23, 24]. The induction of cold response genes starts with the activation of so-called inducer of CBF expression (*ICE*) genes. These genes are MYC-type basic helix–loop–helix transcription factors which bind on MYC recognition sites of C-repeat binding factor (*CBF*) promoters and consequently activate the expression of these genes [25]. The CBF transcription factors are members of the APETALA2/Ethylene response element binding protein (AP2/EREBP) family of DNA-binding proteins [26, 27]. The AP2/EREBP DNA-binding protein domain comprises a structure with three β-strands and one α-helix [28–30]. Furthermore, PKK/RPAGRxKFxETRHP and DSAWR motifs are present, which are typical features of CBF proteins [31]. The CBF transcription factors bind to the C-repeat/dehydration-responsive element (CRT/DRE) and induce the expression of cold-responsive/late embryogenesis–abundant (*COR*/*LEA*) genes [32–34]. The CRT/DRE element is a highly conserved CCGAC sequence in the promoter of cold- and dehydration-responsive genes [35].

The flowering pathway is involved in FT because it contains vernalisation (*VRN*) and photoperiod response (*PPD*) genes that contributed to low temperature acclimatisation [36, 37]. The PPD1 is a member of the PRR (pseudo response regulator) protein family and interacts with CONSTANS (CO) [38]. This family possesses a pseudo-receiver domain and a CONSTANS motif [39, 40]. Downstream the *VRN* genes are localised, i.e. *VRN1, VRN2* and *VRN3*. *VRN1* encodes a MADS-box transcription factor, *VRN2* is similar to a putative zinc finger and a CCT domain and *VRN3* encodes a RAF kinase inhibitor like protein [41–45].

Both pathways, the flowering and the cold response pathway, are connected by the interaction of *VRN1* and *CBF* genes. For example, the *VRN1* gene is able to reduce the transcript levels of *CBF*s and *COR* genes under long day conditions [36].

### Current knowledge about frost tolerance of wheat

Two major FT loci, frost resistance 1 (FR1) and frost resistance 2 (FR2), were identified on the long arm of chromosome 5A of wheat [46, 47]. Zhao et al. [48] described an additional FT Quantitative Trait Locus (QTL) on chromosome 5B in wheat germplasm from central Europe. Due to the importance of cold acclimatisation in winter and spring wheat, the locus FR1 was physically and genetically mapped [49, 50]. However, it is not clear whether FR1 is an independent gene or is based on a pleiotropic effect of *VRN1* [36, 51]. As a consequence of the presence of the A, B and D genome, there are three *VRN1* homologous genes (*VRN-A1*, *VRN-B1* and *VRN-D1*) not having the same impact on vernalisation. Wheat plants with a dominant VRN-A1 allele are spring type and do not need vernalisation for flowering, while the dominant VRN-B1 and VRND1 alleles result also in spring habit, but they are weaker than VRN-A1 [52]. The difference among the spring (dominant *VRN-A1* alleles) and winter varieties (recessive *vrn-A1* alleles) is based on a C/T single nucleotide polymorphism (SNP) in the fourth exon of the *VRN-A1* gene. The winter varieties carrying an ambiguous nucleotide Y (C/T) are more frost tolerant than genotypes carrying the *VRN-B1* or *VRN-D1* allele which both confer higher frost tolerance than spring varieties carrying a C at the respective SNP in *VRN-A1* [2, 53–56].

Plenty of studies identified the FR-A2 locus on chromosome 5A as the most important locus involved in FT in wheat [57–59]. The FR-A2 locus comprises at minimum 11 *CBF* genes and is located approximately 30 cM proximal to *VRN1* [46, 47, 60]. Two independent studies illustrate that *CBF-A3*, which is located in the FR-A2 locus, plays an important role in wheat FT [47, 61]. Knox et al. [62] analysed the FR-A^m^2 locus of diploid *Triticcum monococcum* (A^m^ genome is very similar to the A genome of hexaploid wheat) and identified three *CBFs* (*CBF12*, *CBF14* and *CBF15*) highly associated with FT. Also Vagujfalvi et al. [59] identified *CBF14* and *CBF15* as FT associated in *Triticum monococcum* and Soltesz et al. [63] confirmed this for *Triticum aestivum*. Additionally, Kocsy et al. [64] identified three genes, i.e. *Tacr7* (*Triticum aestivum* cold-regulated 7), *Cab* (calcium-binding EF-hand family protein-like) and *Dem* (Defective embryo and meristems) being differentially expressed during cold hardening in wheat. In addition, on the transcriptome level FT signaling is much more complex. Hundreds to thousands of wheat genes were identified to be significantly up- or downregulated under low temperature [34, 65–69].

The aim of this study was therefore to (i) identity SNPs and indels (insertion-deletion) in genes known to be involved in frost tolerance in wheat and (ii) to conduct a candidate gene based association genetics approach to get information on the effect of respective SNPs and indels on FT.

## Methods

### Plant material and DNA extraction

A diverse set of 235 bread wheat genotypes was used for PCR amplification, amplicon sequencing and association genetics studies. One hundred and seventy-nine cultivars, 48 lines and 8 doubled haploid (DH) lines originating from 28 countries from five continents (Additional file 1: Table S1) were analysed. The association panel was selected based on pre-existing knowledge regarding the reaction to growing conditions during winter time, i.e. high latitude and continental European winter wheat collections as well as Russian and North American cultivars. Furthermore, the core collection of the Institute of Field and Vegetable Crops (IFVCNS), Novi Sad, Serbia [70] and parental lines of Western European hybrid breeding programs were included. For the physical mapping of PCR amplicons to chromosomes and chromosome segments, 21 nulli-tetrasomic lines (NT-lines) and 46 deletion-lines were used [9, 71, 72]. The DNA was extracted at the three leaf stage according to Stein et al. [73].

### Field experiments and phenotypic data analysis

The field experiments were performed in five environments during 2012 (Gatersleben, Germany; Ranzin, Germany; Puskin, Russia; Roshchinskiy, Russia; Novosibirsk, Russia) and 2013 (Gatersleben, Germany; Ranzin, Germany; Puskin, Russia; Roshchinskiy, Russia) and one in 2014 (Novosibirsk, Russia). All 235 genotypes were tested in Gatersleben, Ranzin, Pushkin, and Novosibirsk in a random design in double rows and two replications per genotype. The trial in Roshchinskiy was conducted as a miniplot (2.5m^2^) trial with one replication. FT was evaluated as winter survival in percent (%), i.e. the survival of plants per genotype and plot was measured as a quantitative trait (%) ranging from 0% (all dead) to 100% (all alive) after winter.

To take into account the diversity of the environments with respect to climatic conditions, a co-variable comprising the number of days with an average air temperature under − 15 °C in the period from December 1st to April 30th of each year was calculated. The correlation coefficient r [74] was calculated between the co-variable and FT. Principal component analysis (PCA) was used to get information on the influence of the environment on FT. Correlation coefficient r and PCA were calculated with the JMP® Genomics 5.1 software (SAS, Cary, USA). Data measured at the same location in different years were treated as independent. All data sets that exhibit a deviation described by the second component of PCA analysis, indicate an atypical trait value putatively caused by secondary environmental factors and were discarded. Furthermore, the coefficient of variation (CV; standard deviation divided by arithmetic average) was calculated as well as the variance of each environment and year. The data sets with a very low CV (under 0.15) and/or variance (under 150), were classified as non-representative and were discarded. After editing of the field data, the significance of the influence by environment and genotype was tested by using the analysis of covariance (ANCOVA) and a general linear model (GLM) procedure. Based on this, the Least-Squares means (LS means) were calculated. For all of these analyses the SAS® 9.4 software (SAS, Cary, USA) was used.

### PCR amplification, fragment analysis and re-sequencing

Primer development and testing was conducted according to Babben et al. [9]. Furthermore, the primer assignment was verified based on BLASTs of mRNA and genomic regions of close relatives against the Chinese Spring reference assembly v1.0 using NCBI Megablast. Subsequently, 5 kb upstream and downstream regions were extracted based on the BLAST results and the analysis of the nulli-tertasomic (NT) lines [9]. Primers were aligned to these genomic regions using a free shift alignment with affine gap costs (gap opening = 5, gap elongation = 0.01, match = − 1). If ambiguities were detected at the beginning or the end of exons, primers were manually modified to match the consensus dinucleotides of splice sites, GT and AG. PCR amplification was performed in 20 μl reaction volume (RV) by using two polymerases, i.e. FIREPol**®** DNA polymerase (Solis BioDyne, Tartu, Estonia) and MyTaq™ DNA polymerase (BIOLINE, Luckenwalde, Germany). The master mix for a single PCR reaction comprised 0.4 U FIREPol® DNA Polymerase, 1 x Buffer B, 2.5 mM MgCl_2_ (Solis BioDyne, Tartu, Estonia), 0.2 mM dNTPs (Fermentas, St. Leon-Rot, Germany) and 0.25 pmol primers (Microsynth, Balgach, Switzerland) or 0.4 U MyTaq™ DNA Polymerase, 1 x My Taq Reaction Buffer B (that comprised 1 mM dNTPs and 3 mM MgCl_2_) (BIOLINE, Luckenwalde, Germany) and 0.25 pmol primers. The PCR fragment amplification was conducted in a GeneAmp® PCR System 9700 (Applied Biosystems, Darmstadt, Germany) using various PCR profiles (Additional file 2: Table S2). PCR fragments were separated by agarose gel electrophoresis and analysed using the imaging system Gel Doc™ XR and the Quantity One® 1-D analysis software (4.6.2) (Bio-Rad, Hercules, USA) and subsequently sequenced by the company Microsynth AG (Balgach, Switzerland) using the Sanger sequencing method [75].

### Detection of polymorphisms (SNPs/indels) and haplotypes

The sequencing raw data were edited using Sequencer 5.1 (Gene Codes Corporation, Ann Arbor, USA). Next, the adjusted sequences of each primer pair were aligned by using the Multiple Alignment tool Fast Fourier Transform (MAFFT) [76]. MAFFT parameters were set as default. The polymorphisms between the 235 genotypes were detected automatically via a small multiple sequence alignment (MSA) script (Additional file 3: Data S1) in the free software Java™. Parameters for the polymorphism detection were as follows: polymorphisms between defined bases (A, T, C or G) and ambiguous nucleotides (N) were ignored.. The detected SNPs were used for the identification of haplotypes and components of haplotypes for each candidate gene according to the position in promoter, exon, intron or in the 3` untranslated region (UTR).

### Population structure and kinship calculation

In order to account for population structure effects in association studies, the population structure was estimated based on 249 SNPs, chosen according to the map location and even distribution along the 21 wheat chromosomes [8, 77]. Population structure of wheat accessions was assessed using STRUCTURE v 2.3.3, which is based on a Bayesian model-based clustering algorithm that incorporates admixture and allele correlation models to account for genetic material exchange in populations, resulting in shared ancestry [78]. Five independent runs were performed setting the number of populations (k) from 1 to 10, burn in time and Markov Chain Monte Carlo (MCMC) replication number both to 100,000. The k-value was determined by ln P(D) in STRUCTURE output and an ad hoc statistic Δk based on the rate of change in ln P(D) between successive k-values [79]. Wheat lines with probabilities ≥0.5 were assigned to corresponding clusters. Lines with probabilities < 0.5 were assigned to a mixed group. The population structure plot was constructed by using STRUCTURE PLOT [80] and the Principal coordinate analysis (PCoA) by using the software package DARwin [81]. The kinship (K) matrix was calculated on a modified Roger’s distance [82–84] by using R version 3.2.1 free software [85]. The Roger’s distance was calculated as follows:

1 $$ {d}_w=\frac{1}{\sqrt{2m}}\sqrt{\sum \limits_{i=1}^m\sum \limits_{j=1}^{nj}\Big({p}_{ij}-{q}_{ij}}\Big){}^2 $$

where *pij* and *qij* are allele frequencies of the *j*th allele at the *i*th locus, n number of alleles at the *i*th locus and m number of loci.

### Association genetics analysis

#### SNP and indel association genetics analysis

The SNP/indel association analysis was performed with a set of 235 genotypes by using TASSEL 5.0.9 [86, 87]. Only the SNPs/indels with minor allele frequencies (MAF) > 5% were taken into consideration for analysis. Furthermore, population structure, kinship matrix and phenotypic LS means were included in association studies applying the mixed linear model (MLM) algorithm. The threshold of statistically significant effects was set to 1.30 –log_10_ of *P* (*P-*value of 0.05) according to Li et al. [22, 77] who used this threshold for the analysis of candidate genes at chromosomal regions with high linkage disequilibrium (LD. The LD was calculated via TASSEL 5.0.9 by using the full matrix LD type method with 8064 comparisons after MAF selection.

#### Haplotype association genetics analysis

The haplotype association genetics study was performed by using TASSEL 4.1.20 [86, 87]. The parameters, calculation and significance threshold were the same as used for the SNP and indel association analysis. The same applies to the LD calculation for the genotype groups from Europe, North America and Asia including Australia.

### Sequence analysis

The translation of associated gene sequences into amino acid (AA) sequences, homologue AA sequence search, AA alignments, identification of protein domains and motifs as well as prediction of secondary protein structures were performed using the following software: CLC Main Workbench 7.6 (CLC Bio, Aarhus, Denmark), MAFFT, free software Jalview [88], NCBI (National Center for Biotechnology) protein BLAST [89] and RaptorX [90–92]. The nucleotide sequences were translated into AA sequences via CLC following BLASTn against the NCBI protein database for homologous AA sequence identification. Parameters were set as default. The alignment of initial and homologous AA sequences was performed using CLC and MAFFT. The last step was the identification of domains and motifs and the prediction of the protein structure via RaptorX. For the quality check of the RaptorX results, the uGDT-value, uSeqId-value and *P*-value were taken into account. A uGDT > 50, uSeqId > 30 and a *P*-value less than 1*10^− 3^ are indicators for good quality modelling. Nucleotid divergence rates (dN/dS) between the identified haplotypes and reference sequences of *Triticum aestivum* and related species were analysed using a web-based HyPhy application [93, 94]. Haplotype and reference sequences were used to generate sequence alignments by applying the L-INS-i option in MAFFT [95]. To obtain the best fitting substitution model, the model test application in MEGA-CC was used [96]. The reconstruction of the phylogenetic tree was done with maximum likelihood algorithm and 500 bootstraps in MEGA-CC (using the corresponding model). The resulting protein alignment and the corresponding nucleotide sequences were used to compute codon alignments with Pal2Nal [97]. The codon alignments and the phylogenetic tree were used to compute dN/dS for each variable site using the SLAC method in HyPhy [94].

### *In silico* promoter analysis

Identification of promoter regions and regulatory sites was performed using the internet databases SOGO from the National Institute of Agrobiological Sciences [98, 99] and Softberry [100, 101].

## Results

### Phenotypic data analysis

**Fig. 1:**
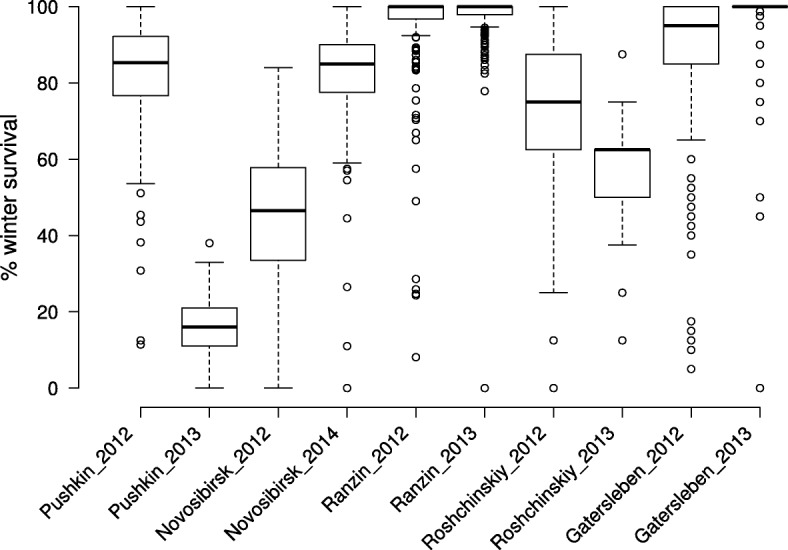
Phenotypic variation at five field locations during two experimental years. Center lines show the medians; box limits indicate the 25th and 75th percentiles as determined by R software; whiskers extend to 5th and 95th percentiles, outliers are represented by dots. *n* > 203

Phenotypic data of five field locations were obtained during 2 years (Fig. 1 and Additional file 4: Figure S1). These phenotypic data sets and the established co-variable (number of days under − 15 °C) are only weakly correlated (*r* = 0.24), indicating that all phenotypic data are not suitable to be used for FT analysis. All the FT scores were transformed using arcsine and Cox-Box (data not shown), but transformations did not result in any improvement. A scatter plot illustrates that the phenotypic data obtained in Pushkin_2013 and Novosibirsk_2014 are not due to FT (Additional file 4: Figure S1). A reason for this may be the very few days under − 15 °C at Pushkin in 2013 (10 days) but a rather low winter hardiness (mean winter survival of 16.2%) due to missing snow coverage; while in Novosibirsk in 2014 a very high number of days under − 15 °C (41 days) but less frost damage was observed due to continuous snow coverage (mean winter survival of 82.6%). The PCA calculation shows that the environments Ranzin_2013, Gatersleben_2013 and Novosibirsk_2014 are separated from the other environments described by the second component (Additional file 5: Figure S2). This indicates that at these locations in the respective years phenotypic data are influenced by additional factors and are not entirely due to differences in FT. An additional characteristic of phenotypic data is the CV and variance (in %) calculation of each environment per year. High CVs (over 0.15) and/or variances (over 150) represent a good phenotypic distribution of all 235 genotypes within the field data sets. Ranzin_2013, Gatersleben_2013 and Novosibirsk_2014 show very low CVs between 0.08 and 0.15 and variances between 54.3 and 149.4. The environment Pushkin_2013 shows a low variance with 51 but a high CV value of 0.44. These values resulted from a very low winter survival with a low standard deviation (Table 1). In conclusion of the phenotypic data analysis, environments Ranzin_2013, Gatersleben_2013, Pushkin_2013 and Novosibirsk_2014 were excluded from further analysis because results are not really related to FT. Out of the data sets Ranzin_2012, Gatersleben_2012, Pushkin_2012, Novosibirsk_2012, Roshchinskiy_2012 and Roshchinskiy_2013 the LS means were calculated (Additional file 1: Table S1).

**Table 1:** Variance and coefficient of variation of frost tolerance data

Location/Year	Size	Missing data	Mean frost tolerance in %	Standard deviation	Variance	Coefficient of variation
Pushkin_2012	235	0	82.40	13.88	192.52	0.17
Pushkin_2013	235	0	16.16	7.14	51.01	0.44
Novosibirsk_2012	235	0	45.60	16.46	271.05	0.36
Novosibirsk_2014	235	0	82.58	12.22	149.42	0.15
Ranzin_2012	235	0	95.20	13.31	166.12	0.14
Ranzin_2013	235	0	97.67	8.19	54.27	0.08
Roshchinskyiy_2012	235	32	69.33	22.64	512.53	0.33
Roshchinskiy_2013	235	4	56.55	13.68	187.24	0.24
Gatersleben_2012	235	0	85.89	22.14	490.01	0.26
Gatersleben_2013	235	0	97.18	9.12	83.21	0.09

#### Candidate gene polymorphisms

**Fig. 2:**
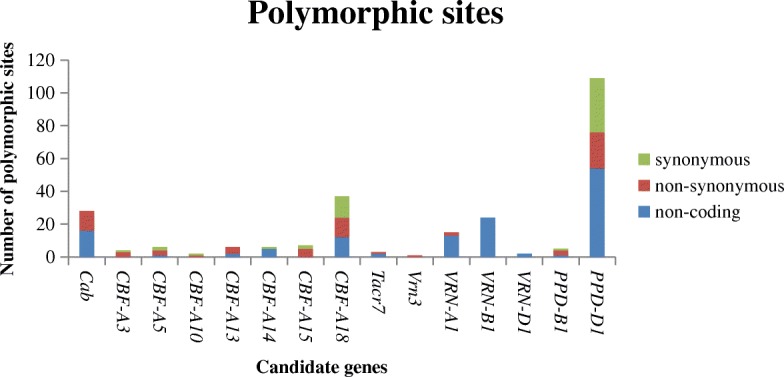
Number of synonymous, non-synonymous and non-coding mutations per candidate gene

A set of 65 specific primer pairs from the previous study [9] was BLASTed against the IWGSC RefSeq allowing the identification of 39 primer combinations that cover the full length of 19 genes and their structural analyses. An optimised set of 39 primer pairs was used for PCR amplification, amplicon sequencing and association genetics studies. No exact position could be determined for the first forward primer of *PPD-B1* and the third forward primer of *VRN-D2*, since BLASTN revealed seven and five matches, respectively. For all other primers an exact position was determined. In addition, an unassigned scaffold was identified for *PPD-B1* located on chromosome 2B (Additional file 6: Figure S3). The total re-sequenced length of candidate genes was 13.3 Kbp coding DNA sequence (CDS) and 43.76 Kbp genomic lengths, with a CDS/genomic length ratio of 0.30. In total, a sequence length of 33.5 Kbp was analysed. In our set of 235 genotypes, the 15 candidate genes *Cab*, *CBF-A3*, *CBF-A5*, *CBF-A10*, *CBF-A13*, *CBF-A14*, *CBF-A15*, *CBF-A18*, *Tacr7*, *VRN-B3*, *VRN-A1*, *VRN-B1*, *VRN-D1*, *PPD-B1* and *PPD-D1* were polymorphic and four (*CBF-D1*, *Dhn1*, *VRN2* and *Dem*) (Table 2) were monomorphic. In total 254 polymorphic sites, i.e. 221 SNPs and 33 indels were identified. The SNP number per gene ranged from 0 to 97 and the indel number from 0 to 12. Over all genes, 42 polymorphic sites in promoter regions, 64 in introns, 25 in 3` UTRs and 123 in exons were identified. Out of the 254 polymorphic sites, 131 were located in non-coding regions, and 54 synonymous and 69 non-synonymous polymorphic sites were identified (Fig. 2). The number of haplotypes ranged between two and six and the haplotype diversity (Hd) between 0.07 and 0.68 (Table 3).

**Table 2:** List of analysed FT candidate genes, sequence length, number of specific PCR fragments and detected mutations

Gene	Chr. position	Number of specific PCR fragments	Sequence length in bp	CDS length in bp	Gene length in bp	CDS/gene length ratio	Detected mutations	Number of polymorphic sites
*CBF-D1*	5D	1	709	639	639	1.00	no	0
*CBF-A3*	5A	1	790	741	741	1.00	yes	4
*CBF-A5*	7A	1	1027	633	633	1.00	yes	6
*CBF-A10*	5A	1	867	720	720	1.00	yes	2
*CBF-A13*	5A	1	855	720	720	1.00	yes	6
*CBF-A14*	5A	2	610/748	639	639	1.00	yes	6
*CBF-A15*	5A	1	786	726	726	1.00	yes	7
*CBF-A18*	6A	1	1045	738	738	1.00	yes	37
*Dhn1*	5D	1	936	420	638	0.66	no	0
*VRN-A1*	5A	4	1039/1097/621/770	735	11,414	0.06	yes	15
*VRN-B1*	5B	5	600/817/662/467/1077	735	5498	0.13	yes	24
*VRN-D1*	5D	4	814/1359/1429/705	735	11,550	0.06	yes	2
*VRN-D2*	4D	2	976/534	639	1650	0.39	no	0
*VRN-B3*	7B	2	1602/994	534	1258	0.42	yes	1
*Cab*	5A	1	728	n.a.	n.a.	n.a.	yes	28
*Dem*	6B/6D	1	616	n.a.	n.a.	n.a.	no	0
*Tacr7*	2B	1	765	n.a.	n.a.	n.a.	yes	3
*PPD-B1*	2B	6	1600/954/927/571/773/378	1995	3053	0.65	yes	5
*PPD-D1*	2D	3	726/1094/966	1983	3141	0.63	yes	109
total		39	34,034	13,332	43,758	0.30		255

*Chr.* chromosome, *bp* base pairs, *CDS* coding DNA sequence, *n.a.* not available

**Table 3:** Polymorphic candidate genes, location of polymorphisms, amino acid change and haplotype diversity

Gene	Chr.	Acces-sions	Poly-morphic sites	SNPs	in-dels	Promotor	Intron	Exon	3` UTR	Non-coding	Non-syno-nymous	Syno-nymous	Haplo-types	Hd
*CBF-A3*	5A	235	4	4	0	0	0	4	0	0	3	1	2	0.30
*CBF-A5*	7A	235	6	5	1	1	0	5	0	1	3	2	4	0.54
*CBF-A10*	5A	235	2	2	0		0	2	0	0	1	1	2	0.30
*CBF-A13*	5A	235	6	4	2	1	0	5	0	2	4	0	3	0.30
*CBF-A14*	5A	235	6	5	1	5	0	1	0	5	0	1	3	0.30
*CBF-A15*	5A	235	7	6	1	0	0	7	0	0	5	2	2	0.30
*CBF-A18*	6A	235	37	34	3	1	0	25	11	12	12	13	3	0.20
*VRN-A1*	5A	235	15	10	5	7	6	2	0	13	2	0	6	0.19
*VRN-B1*	5B	235	24	21	3	20	4	0	0	24	0	0	5	0.07
*VRN-D1*	5D	235	2	2	0	0	1	0	1	2	0	0	3	0.14
*VRN-B3*	7B	235	1	1	0	0	0	1	0	0	1	0	3	0.50
*Cab*	5A	235	28	24	4	5	0	12	11	16	12	0	6	0.64
*Tacr7*	2B	235	3	2	1	0	0	1	2	2	1	0	3	0.40
*PPD-B1*	2B	235	5	5	0	1	0	4	0	1	3	1	6	0.37
*PPD-D1*	2D	235	109	97	12	1	53	55	0	54	22	33	6	0.68
total			255	222	33	42	64	124	25	132	69	54	58	

*Chr.* chromosome, *SNP* single nucleotide polymorphism, *indel* insertion-deletion, *UTR* untranslated region, *Hd* Haplotype Diversity

### Population structure and kinship

**Fig. 3:**
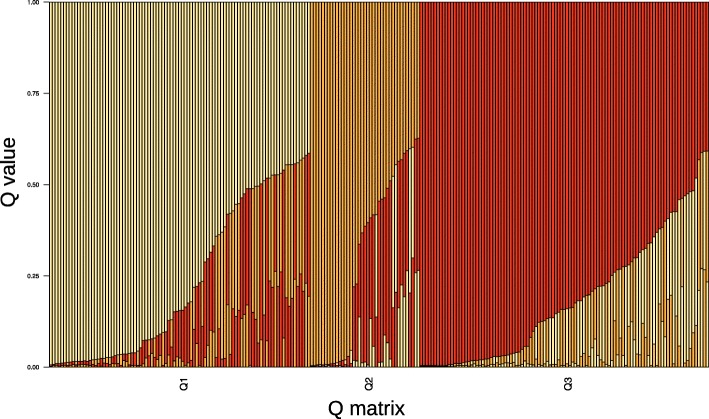
Population structure of 235 wheat cultivars based on 249 SNPs. Each individual is represented by a single vertical line that is partitioned into Q colored segments (Q = 3) in the x-axis. The y-axis illustrated the Q-value

Genetic profiles were obtained by using the ILLUMINA Infinium iSelect 90 k wheat chip. These data were kindly provided by the Dr. A. Börner from IPK Gatersleben and will be used for GWAS analysis in an additional paper. As an outcome of the STRUCTURE analysis based on 249 SNPs covering the whole genome, k = 3 was the most probable number of sub-populations mostly corresponding to the origin of genotypes. The neighbour-joining tree revealed three sub-populations i.e. North American, Russian, and North and Central European genotypes. This population structure resembled a tight association to the origin of the genotypes analysed. In the first sub-population, accessions from European countries, subdivided into two groups, i.e. North and Central European genotypes, are included. Accessions from Canada, Mexico and USA were predominantly in the second sub-population, and the third sub-population contained predominantly accessions from Russia and Kazakhstan. The population structure is shown in Fig. 3 and Additional file 7: Figure S4. The STRUCTURE membership coefficients and the modified Roger’s distance revealed a high degree of admixture in a large number of accessions. Therefore, many accessions could not be classified to main groups, because their genomes represent a mixture of the main groups. This may be due to germplasm exchange and it use in breeding programs.

### SNP/indel association analysis

**Fig. 4:**
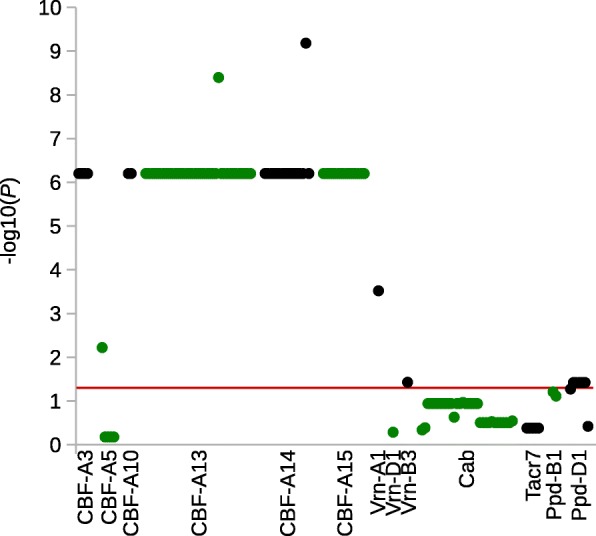
Manhattan plot of SNPs/indels in candidate genes for FT. The -log10 (*P*-values) from the association analysis are plotted against the respective candidate gene. The red horizontal line indicates the significance threshold at *P* < 0.05. For better visualisation, the successive genes are shown in alternating black and green colours

**Fig. 5:**
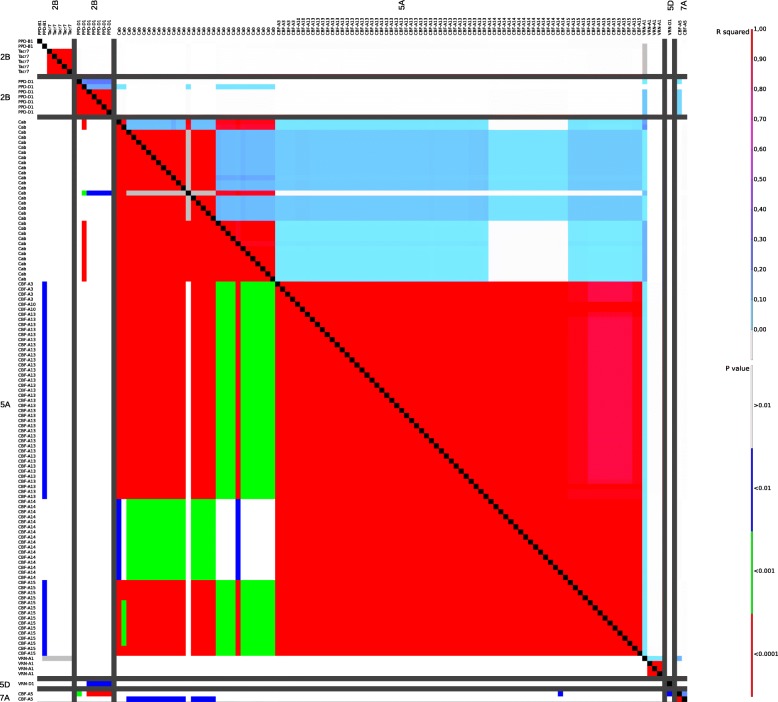
LD plot of all used SNPs and indels for association analysis. On the left side and at the top the genes and chromosomes with polymorphic sites are shown. Each coloured square in the below triangle represents the intensity of LD expressed by *P*-values for each pairwise comparison between polymorphic sites. Each coloured square in the top triangle represents the intensity of LD expressed by r^2^ for each pairwise comparison between polymorphic sites. On the right side the legend for r^2^ values and *P*-values are shown

The association analysis was performed using 254 polymorphic sites identified in 15 candidate genes. After MAF selection, 58 polymorphic sites, i.e. 46 SNPs and 12 indels, from 13 candidate genes (*Cab*, *CBF-A3*, *CBF-A5*, *CBF-A10*, *CBF-A13*, *CBF-A14*, *CBF-A15*, *Tacr7*, *VRN-B3*, *VRN-A1*, *VRN-D1*, *PPD-B1* and *PPD-D1*) were included in further analysis. In the SNP/indel association, 27 statistically significant (*P* < 0.05) polymorphic sites (21 SNPs and six indels) in six candidate genes were identified (Table 4). Four SNPs and two indels were located in the promoter region of *CBF-A5*, *CBF-A13* and *CBF-A14*. The remaining 17 SNPs and four indels were located in exon regions of five *CBFs*, *PPD-D1*, *VRN-A1* and *VRN-B3*. 11 of these SNPs are non-synonymous. Six associated genes were located on wheat chromosome 5A and one each on chromosome 2D, 7A and 7B. Allelic effects of significantly associated polymorphic sites on FT ranged from 0.11% to 15.01 (Fig. 4, Table 4, Additional file 8: Table S3). With an LD of r^2^ = 0.92 to 1, the statistical significances of associated SNPs and indels from the five *CBF* genes on chromosome 5A (*CBF-A3*, *CBF-A10*, *CBF-A13*, *CBF-A14* and *CBF-A15*) is very high. The LD plot of all used SNPs/indels for association calculation is shown in Fig. 5.

**Table 4:** Statistics of significantly associated SNPs and indels

Gene/Polymorphism name	CDS and promotor site	*P-*value	-Log of *P*	Polymorphism			Effect in %			Observations^a^		
CBF-A3_SNP1	222	6.28E-10	9.20	A	C		15.01	0.00		193	42	
CBF-A3_SNP2	263	6.28E-10	9.20	C	G		15.01	0.00		193	42	
CBF-A3_SNP3	367	6.28E-10	9.20	A	G		15.01	0.00		193	42	
CBF-A3_SNP4	407	6.28E-10	9.20	C	T		15.01	0.00		193	42	
CBF-A5_indel1	−83	6.00E-3	2.22	C	–		−4.52	0.00		141	84	
CBF-A10_SNP1	471	6.28E-10	9.20	C	T		15.01	0.00		193	42	
CBF-A10_SNP2	518	6.28E-10	9.20	G	C		15.01	0.00		193	42	
CBF-A13_SNP1	−11	6.28E-10	9.20	T	C		15.01	0.00		193	42	
CBF-A13_indel3	19	6.28E-10	9.20	T	–		15.01	0.00		193	42	
CBF-A13_SNP2	92	6.28E-10	9.20	G	A		15.01	0.00		193	42	
CBF-A13_indel2	133	6.28E-10	9.20	–	C		15.01	0.00		193	42	
	134	6.28E-10	9.20	–	G		15.01	0.00		193	42	
	135	6.28E-10	9.20	–	T		15.01	0.00		193	42	
	136	6.28E-10	9.20	–	G		15.01	0.00		193	42	
	137	6.28E-10	9.20	–	C		15.01	0.00		193	42	
	138	6.28E-10	9.20	–	G		15.01	0.00		193	42	
	139	6.28E-10	9.20	–	G		15.01	0.00		193	42	
	140	6.28E-10	9.20	–	C	A	15.22	0.00	6.75	193	41	1
	141	6.28E-10	9.20	–	G		15.01	0.00		193	42	
	142	6.28E-10	9.20	–	C		15.01	0.00		193	42	
	143	6.28E-10	9.20	–	A		15.01	0.00		193	42	
	144	6.28E-10	9.20	–	G		15.01	0.00		193	42	
	145	6.28E-10	9.20	–	G		15.01	0.00		193	42	
	146	6.28E-10	9.20	–	G		15.01	0.00		193	42	
	147	6.28E-10	9.20	–	G		15.01	0.00		193	42	
	148	6.28E-10	9.20	–	C		15.01	0.00		193	42	
	149	6.28E-10	9.20	–	A		15.01	0.00		193	42	
	150	6.28E-10	9.20	–	A		15.01	0.00		193	42	
	151	6.28E-10	9.20	–	C		15.01	0.00		193	42	
	152	6.28E-10	9.20	–	G		15.01	0.00		193	42	
	153	6.28E-10	9.20	–	C		15.01	0.00		193	42	
	154	6.28E-10	9.20	–	G		15.01	0.00		193	42	
	155	4.00E-10	9.40	–	G		15.01	0.00		193	42	
	156	6.28E-10	9.20	–	G		15.01	0.00		193	42	
	157	6.28E-10	9.20	–	G		15.01	0.00		193	42	
	158	6.28E-10	9.20	–	C		15.01	0.00		193	42	
	159	6.28E-10	9.20	–	G		15.01	0.00		193	42	
	160	6.28E-10	9.20	–	G		15.01	0.00		193	42	
	161	6.28E-10	9.20	–	T		15.01	0.00		193	42	
	162	6.28E-10	9.20	–	G		15.01	0.00		193	42	
	163	6.28E-10	9.20	–	G		15.01	0.00		193	42	
	164	6.28E-10	9.20	–	G		15.01	0.00		193	42	
CBF-A13_SNP3	294	6.28E-10	9.20	C	A		15.01	0.00		193	42	
CBF-A14_indel1	−506	6.28E-10	9.20	G	–		15.01	0.00		193	42	
	− 505	6.28E-10	9.20	T	–		15.01	0.00		193	42	
	− 504	6.28E-10	9.20	G	–		15.01	0.00		193	42	
	−503	6.28E-10	9.20	A	–		15.01	0.00		193	42	
	− 502	6.28E-10	9.20	G	–		15.01	0.00		193	42	
	−501	6.28E-10	9.20	T	–		15.01	0.00		193	42	
	−500	6.28E-10	9.20	G	–		15.01	0.00		193	42	
	− 499	6.28E-10	9.20	T	–		15.01	0.00		193	42	
	−498	6.28E-10	9.20	G	–		15.01	0.00		193	42	
	− 497	6.28E-10	9.20	A	–		15.01	0.00		193	42	
	−496	6.28E-10	9.20	G	–		15.01	0.00		193	42	
	− 495	6.28E-10	9.20	T	–		15.01	0.00		193	42	
CBF-A14_SNP1	− 449	6.28E-10	9.20	T	C		15.01	0.00		193	42	
CBF-A14_SNP2	− 158	6.28E-10	9.20	T	C		15.01	0.00		193	42	
CBF-A14_SNP3	−53	6.57E-10	9.18	C	G		15.01	0.00		193	42	
CBF-A14_SNP4	576	6.28E-10	9.20	G	A		15.01	0.00		193	42	
CBF-A15_SNP1	84	6.28E-10	9.20	C	T		15.01	0.00		193	42	
CBF-A15_SNP2	243	6.28E-10	9.20	T	C		15.01	0.00		193	42	
CBF-A15_SNP3	293	6.28E-10	9.20	C	T		15.01	0.00		193	42	
CBF-A15_SNP4	397	6.28E-10	9.20	G	A		15.01	0.00		193	42	
CBF-A15_indel1	503	6.28E-10	9.20	T	–		15.01	0.00		193	42	
	504	6.28E-10	9.20	C	–		15.01	0.00		193	42	
	505	6.28E-10	9.20	G	–		15.01	0.00		193	42	
	506	6.28E-10	9.20	T	–		15.01	0.00		193	42	
	507	6.28E-10	9.20	C	–		15.01	0.00		193	42	
	508	6.28E-10	9.20	G	–		15.01	0.00		193	42	
	509	6.28E-10	9.20	T	–		15.01	0.00		193	42	
	510	6.28E-10	9.20	C	–		15.01	0.00		193	42	
	511	6.28E-10	9.20	G	–		15.01	0.00		193	42	
CBF-A15_SNP5	694	6.28E-10	9.20	A	G		15.01	0.00		193	42	
CBF-A15_SNP6	716	6.28E-10	9.20	C	G		15.01	0.00		193	42	
PPD-D1_indel1	1266	3.77E-2	1.42	–	C		3.96	0.00		68	166	
	1267	3.77E-2	1.42	–	G		3.96	0.00		68	166	
	1268	3.77E-2	1.42	–	T		3.96	0.00		68	166	
	1269	3.77E-2	1.42	–	C		3.96	0.00		68	166	
	1270	3.77E-2	1.42	–	G		3.96	0.00		68	166	
VRN-A1_SNP1	349	3.03E-4	3.52	C	T	Y	0.00	1.35	0.11	24	2	209
VRN-B3_SNP1	19	3.76E-2	1.43	C	G		3.05	0.00		105	128	

*CDS* coding DNA sequence, *P* probability, *Log* logarithm

^a^number of genotypes per allele

### Linkage disequilibrium (LD) and germplasm origin

Out of 235 studied genotypes, 116 originate from Europe, 38 from North America and 81 from Asia. After MAF selection, 51 polymorphic sites were identified in the genotypes from Europe, 88 in those derived from North America and 25 in Asian genotypes. All three different sub-populations show that the *CBF* genes on chromosome 5A are in a very high LD, and are separated from other 5A located genes, i.e. *VRN-A1* and *Cab*. All non-5A candidate genes show a low LD to each other in all sub-populations.

### Haplotype association analysis

**Fig. 6:**
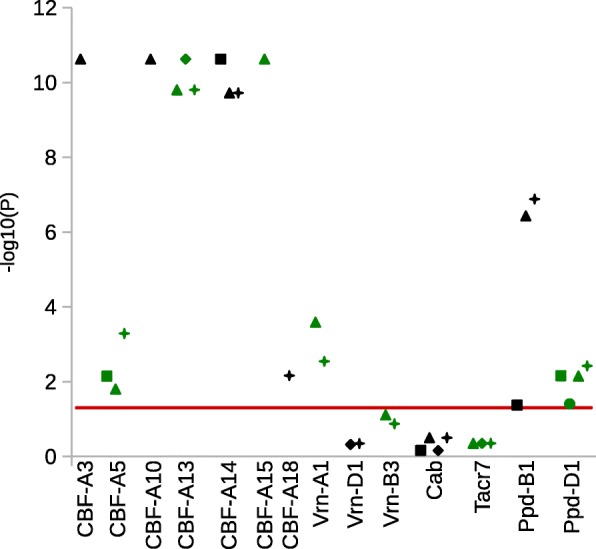
Manhattan plot of haplotypes based on FT candidate genes. The -log10 (*P*-values) from the association analysis are plotted against candidate gene. The red horizontal line indicates the significance threshold at *P* < 0.05. The squares show the promotor, triangle the exon, circles the intron, diamonds the 3’ UTR and stars the whole haplotypes. For better visualisation, the successive genes are shown in alternating black and green colours

A set of 44 haplotypes and components of haplotypes from 15 candidate genes were identified. After MAF selection, 32 haplotypes (six promoter haplotypes, 12 exon haplotypes, one intron haplotype, three 3` UTR haplotypes and 10 whole gene haplotypes) in 14 candidate genes (*Cab*, *CBF-A3*, *CBF-A5*, *CBF-A10*, *CBF-A13*, *CBF-A14*, *CBF-A15*, *CBF-A18*, *Tacr7*, *VRN-B3*, *VRN-A1*, *VRN-D1*, *PPD-B1* and *PPD-D1*) were used for haplotype analysis. In the haplotype association analysis, 22 haplotypes (five promoter haplotypes, nine exon haplotypes, one intron haplotype and seven whole gene haplotypes) in ten candidate genes revealed significant associations (*P* < 0.05) (Table 5). *CBF-A18* is the only exon haplotype which is not significantly associated to FT. The same applies for promoter haplotypes of *CBF-A5*, *CBF-A13*, *CBF-A14*, *PPD-B1* and *PPD-D1*, intron haplotype of *PPD-D1* and the whole gene haplotypes of *CBF-A5*, *CBF-A13*, *CBF-A14*, *CBF-A18*, *PPD-B1*, *PPD-D1* and *VRN-A1*. The associated genes are located on chromosome 2B (*PPD-B1*), 2D (*PPD-D1*), 5A (*CBF-A3*, *CBF-A10*, *CBF-A13*, *CBF-A14*, *CBF-A15*, *VRN-A1*), 6A (*CBF-A18*) and 7A (*CBF-A5*). The allelic effects of associated haplotypes for winter survival ranged from 0.68% to 33.95 (Fig. 6, Table 5, Additional file 8: Table S3).

**Table 5:** Statistics of haplotypes significantly associated to FT

Haplotype name	Chr.	*P-*value	-Log of *P*	Haplotype						FT effect of haplotypes in %						Observations^a^					
CBF-A3 ex.	5A	2.37E-11	10.63	1	2					15.01	0.00					193	42				
CBF-A5 pro.	7A	7.17E-3	2.14	1	2					4.44	0.00					82	143				
CBF-A5 ex.	7A	1.58E-2	1.80	1	2	3				19.51	18.09	0.00				204	28	3			
CBF-A5	7A	5.16E-4	3.29	1	2	3	4			24.18	19.29	18.88	0.00			55	27	140	3		
CBF-A10 ex.	5A	2.37E-11	10.63	1	2					15.01	0.00					193	42				
CBF-A13 ex.	5A	1.57E-10	9.80	1	2	3				15.22	0.00	8.47				193	41	1			
CBF-A13 pro.	5A	2.37E-11	10.63	1	2					15.01	0.00					193	42				
CBF-A13	5A	1.57E-10	9.80	1	2	3				15.22	0.00	8.47				193	41	1			
CBF-A14 pro.	5A	2.37E-11	10.63	1	2					15.01	0.00					193	42				
CBF-A14 ex.	5A	1.90E-10	9.72	1	2	3				17.01	2.11	0.00				193	40	2			
CBF-A14	5A	1.90E-10	9.72	1	2	3				17.01	2.11	0.00				193	40	2			
CBF-A15 ex.	5A	2.37E-11	10.63	1	2					15.01	0.00					193	42				
CBF-A18	6A	6.88E-3	2.16	1	2	3				9.32	6.66	0.00				209	9	17			
PPD-B1 pro.	2B	4.23E-2	1.37	1	2					5.50	0.00					213	22				
PPD-B1 ex.	2B	3.69E-7	6.43	1	2	3	4			18.97	0.00	28.28	33.39			3	5	207	20		
PPD-B1	2B	1.31E-7	6.88	1	2	3	4	5		19.03	0.00	33.95	29.07	22.63		3	5	20	185	22	
PPD-D1 pro.	2D	6.95E-3	2.16	1	2	3				14.38	10.75	0.00				141	80	4			
PPD-D1 in.	2D	3.93E-2	1.41	1	2	3				18.66	19.56	0.00				162	69	3			
PPD-D1 ex.	2D	7.05E-3	2.15	1	2	3				21.80	18.18	0.00				68	163	3			
PPD-D1	2D	3.78E-3	2.42	1	2	3	4	5	6	21.47	3.77	19.28	16.13	0.00	20.55	68	3	69	90	3	1
VRN-A1 ex.	5A	2.57E-4	3.59	1	2	3	4			0.00	0.68	13.12	11.89			9	15	2	209		
VRN-A1	5A	2.87E-3	2.54	1	2	3	4	5	6	14.13	15.90	2.26	15.30	0.00	8.90	207	2	13	2	6	1

*Chr.* chromosome, *ex.* exon, *pro.* Promotor, *in.* intron, *P* probability, *Log* logarithm, *FT* frost tolerance

^a^number of genotypes per allele

### In silico sequence analysis

#### C-repeat binding factors (CBFs)

**Fig. 7:**
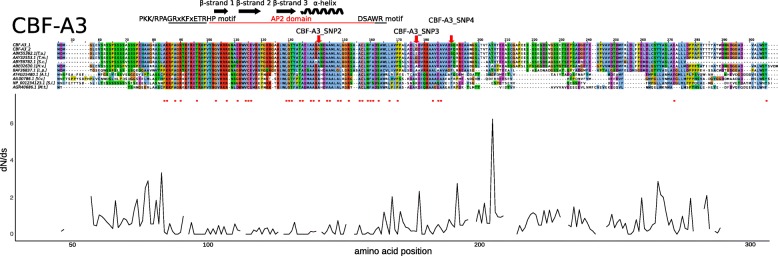
Amino acid alignment and nucleotide divergence rates (dN/dS) of *CBF-A3* gene and homologous amino acid sequences. Shown are alignments of two haplotype AA sequences of CBF-A3 and nine homologue plant AA sequences. The numbers above the alignment indicate the sites of AAs. The black line above the alignment illustrates the PKK/RPAGRxKFxETRHP and DSAWR motif, the red line the AP2 domain, the black arrows the β-strands and the black spirals the α-helices. The red arrows label the AA changes based on significantly associated SNPs or indels. The plot below the alignment shows the nucleotide divergence rates (dN/dS). The black line describes the dN/dS ratio. The red dots between alignment and plot indicate sites with significant negative selection (*P* < 0.05)

To validate and annotate obtained sequences of candidate genes, an in silico analysis was performed. NCBI protein BLASTX of CBF AA sequences results in nine homologues from five related and four unrelated species (Additional file 9: Table S4). For the CBFs (CBF-A3, CBF-A5, CBF-A10, CBF-A13, CBF-A14, CBF-A15, CBF-A18) that showed a significant association to FT, the AP2/EREBP transcription factor domain was identified, which is predicted to encode a protein structure with three β-strands and one α-helix (2gcc). The parameters of protein structure modelling are shown in Table 6. The CBF-A13_1 haplotype protein shows a low *P*-value of 4.79 × 10^− 4^, and a high uGDT of 51, and a uSeqId of 15 extent. Protein structure of CBF-A13_2 could not be predicted. In all remaining cases protein structure models were of high quality. Additionally, the PKK/RPAGRxKFxETRHP and DSAWR motifs were identified, which are typical features of CBF proteins. The AA sequences between two CBF-A3 haplotypes revealed three AA substitutions. The CBF-A3_SNP2 [C/G] on CDS site 263 leads to an Ala/Gly change, CBF-A3_SNP3 [A/G] on CDS site 367 to a Ser/Gly change and CBF-A3_SNP4 [C/T] on CDS site 407 to a Ser/Phe change. The AA change of CBF-A3_SNP2 is located in the α helix of the AP2 domain. The CBF-A3_SNP3 and CBF-A3_SNP4 is located upstream of the identified domains/motifs. The AA site of CBF-A3_SNP2 is significant for negative selection (Fig. 7). The AA haplotypes of CBF-A5 show three AA changes, which are not significantly associated in the SNP/indel analysis. In contrast CBF-A5_SNP2 [C/A] resulted in an Arg/Ser and CBF-A5_SNP4 [A/G] in a His/Arg change which are located in the β strand 3 or the α helix of the AP2 domain, respectively. Additionally, CBF-A5_SNP4 site underlies negative selection (Additional file 10: Figure S5). The AA haplotypes of CBF-A10 show an AA change of Gly/Ala based on CBF-A10_SNP2 [G/C] on CDS site 518. This substitution is upstream of the identified domains/motifs and at a site not showing positive or negative selection (Additional file 11: Figure S6). The haplotypes of CBF-A13 show completely different AA sequences. The single bp deletion on CDS site 19 of CBF-A13_indel results in a frame shift that changes every AA from the seventh AA onwards. Furthermore, a stop codon at position 74 AAs was detected. The haplotype one shows a 32 bp deletion at CDS site 133 to 164 (CBF-A13_indel2). This deletion is localised in the AP2 domain and causes a stop codon after 135 AAs. In summary, haplotype one of CBF-A13 shows a higher similarity to homologue AA sequences of related species and the AP2 domain than haplotype two. Due to the short AA sequences of both CBF-A13 haplotypes and low similarity to homologue AA sequences, no assumptions about positive or negative selection can be made (Additional file 12: Figure S7).

**Table 6:** Protein structure modelling of associated genes

Protein haplotypes	*P*-value	uGDT (GDT)	uSeqId (SeqId)	Score	Domain/motif	Literature
CBF-A3_1	3.03E^−4^	71 (62)	30 (26)	51	2gcc (AP2)	[28]
CBF-A3_2	1.52E^−4^	71 (61)	29 (25)	51	2gcc (AP2)	[28]
CBF-A5_1	3.22E^−4^	69 (67)	30 (29)	51	2gcc (AP2)	[28]
CBF-A5_2	5.75E^−4^	56 (54)	30 (29)	54	2gcc (AP2)	[28]
CBF-A5_3	9.71E^−4^	57 (56)	29 (28)	56	2gcc (AP2)	[28]
CBF-A10_1	2.78E^−4^	70 (66)	31 (29)	51	2gcc (AP2)	[28]
CBF-A10_2	2.88E^−4^	71 (67)	31 (29)	50	2gcc (AP2)	[28]
CBF-A13_1	4.79E^−4^	51 (38)	15 (11)	36	2gcc (AP2)	[28]
CBF-A13_2	n.a.	n.a.	n.a.	n.a.	n.a.	n.a.
CBF-A14_1	1.97E^−4^	71 (69)	29 (28)	52	2gcc (AP2)	[28]
CBF-A14_2	1.97E^−4^	71 (69)	29 (28)	52	2gcc (AP2)	[28]
CBF-A15_1	3.80E^−4^	70 (66)	29 (27)	50	2gcc (AP2)	[28]
CBF-A15_2	2.95E^−4^	70 (70)	29 (29)	49	2gcc (AP2)	[28]
CBF-A18_1	1.33E^−3^	55 (49)	29 (26)	52	2gcc (AP2)	[28]
CBF-A18_2	8.72E^− 4^	55 (49)	29 (26)	54	2gcc (AP2)	[28]
PPD-B1	1.24E^− 4^	105(62)	32(19)	130	3t6k (putative response regulator domain)	n.a.
	8.74E^−5^	101(60)	27(16)	134	3jte (response regulator receiver domain)	[111]
PPD-B2	1.21E^−4^	104(61)	32(19)	129	3t6k (putative response regulator domain)	n.a.
	8.12E^−5^	101(59)	27(16)	134	3jte (response regulator receiver domain)	[111]
PPD-B3	1.06E^−4^	103(60)	31(18)	129	3t6k (putative response regulator domain)	n.a.
	7.21E^−5^	102(59)	27(16)	134	3jte (response regulator receiver domain)	[111]
PPD-B4	1.28E^−4^	104(61)	32(19)	130	3t6k (putative response regulator domain)	n.a.
	8.98E^−5^	102(59)	27(16)	134	3jte (response regulator receiver domain)	[111]
PPD-D1_1	1.05E^−4^	104 (47)	32 (15)	131	3t6k (putative response regulator domain)	n.a.
	7.11E^−5^	102 (46)	27 (12)	136	3jte (response regulator receiver domain)	[111]
PPD-D1_2	1.14E^−4^	105 (63)	32 (19)	130	3t6k (putative response regulator domain)	n.a.
PPD-D1_3	1.16E^−4^	104(62)	32 (19)	129	3t6k (putative response regulator domain)	n.a.
	8.07E^−5^	101 (60)	27 (16)	134	3jte (response regulator receiver domain)	[111]
VRN-A1_1	8.02E^− 3^	84 (83)	39 (39)	84	4ox0 (K domain)	[110]
	1.02E^−4^	78 (103)	38 (50)	53	3kov (MADS domain/I domain)	[112]
	1.30E^−4^	75 (98)	38 (50)	52	1egw (MADS domain/I domain)	[113]
VRN-A1_2	8.47E^−3^	83 (82)	39 (39)	84	4ox0 (K domain)	[110]
	7.90E^−5^	73 (103)	38 (50)	54	3kov (MADS domain/I domain)	[112]
	9.11E^−5^	74 (98)	38 (50)	53	1egw (MADS domain/I domain)	[113]
VRN-A1_3	7.95E^−3^	84 (83)	38 (38)	84	4ox0 (K domain)	[110]
	7.06E^−5^	78 (103)	38 (50)	54	3kov (MADS domain/I domain)	[112]
	1.08E^−4^	75 (99)	38 (50)	53	1egw (MADS domain/I domain)	[113]
VRN-B3_1	7.92E-^13^	154 (87)	147 (83)	166	3axy (Hd3A protein)	[104]
VRN-B3_2	1.66E-^12^	154 (87)	148 (84)	168	3axy (Hd3A protein)	[104]

*P*: probability, uGDT: unnormalized GDT (Global Distance Test) score, GDT: calculated as uGDT divided by the protein (or domain) length and multiplied by a 100, uSeqID: number of identical residues in the alignment, SeqID: uSeqID normalized by the protein (or domain) sequence length and multiplied by 100, n.a.: not available

**Fig. 8:**
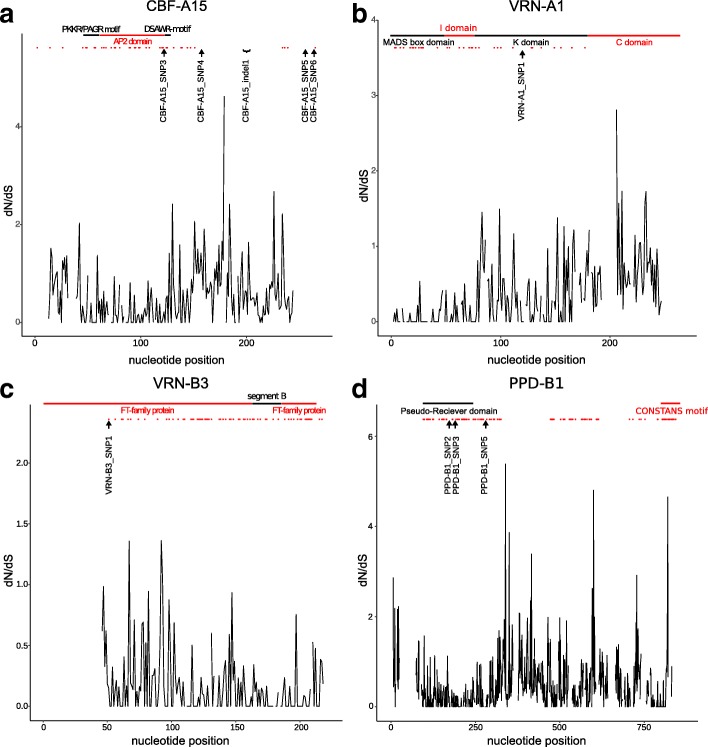
Nucleotide divergence rates (dN/dS) of *CBF-A15, VRN-A1*, *VRN-B3* and *PPD-B1* genes and homologue amino acid sequences. **a** Nucleotide divergence rates (dN/dS) between the identified haplotypes of *CBF-A15*, reference sequence of *T. aestivum* and eight homologous plant AA sequences. The black line on top indicates the PKK/RPAGRxKFxETRHP motif and DSAWR, the red line the AP2 domain. The black arrows and clamp label the position of AA changes based on significantly associated SNPs and indels. The black line shows the dN/dS ratio. The red dots illustrate sites underlying significant negative selection (*P* < 0.05). **b** Nucleotide divergence rates (dN/dS) between the identified haplotypes of *VRN-A1*, reference sequence of *T. aestivum* and eight homologous plant AA sequences. The black line on top indicates the MADS box and K domain, the red line the I and C domain. The black arrow labels the position of AA change based on significantly associated SNPs. The black line describes the dN/dS ratio. The red dots indicate sites with significant negative selection (*P* < 0.05). **c** Illustrated are nucleotide divergence rates (dN/dS) between the identified haplotypes of *VRN-B3*, reference sequence of *T. aestivum* and eight homologous plant AA sequences. The black line on top indicatesthe segment B, the red line the flowering time (FT)-family protein. The black arrow labels the position of AA change based on significant associated SNPs. The black line describes the dN/dS ratio. The red dots illustrate sites with significant negative selection (*P* < 0.05). **d** Nucleotide divergence rates (dN/dS) between the identified haplotypes of *PPD-B1*, reference sequence of *T. aestivum* and eight homologous plant AA sequences. The black line on top indicates the Pseudo-Reciver domain, the red line the CONSTANS motif. The black arrows label the position of AA changes significantly associated SNPs. The black line describes the dN/dS ratio. The red dots indicate sites with significant negative selection (*P* < 0.05)

The AA analysis of two CBF-A15 haplotypes revealed four AA changes and one AA indel. A nine-nucleotide insertion within the coding region (sites 503 to 511) resulted in three additional Ser residues in the haplotype with CBF_indel. The AA change Ala/Val caused by CBF-A15_SNP3 [C/T] on CDS site 293 is located in the AP2 domain. The CBF-A15_SNP4 [G/A] on CDS site 397 resulted in an Ala/Thr AA change. The last AA changes are Ser/Gly caused by CBF-A15_SNP5 [A/G] on CDS site 694 and Ser/Trp caused by CBF-A15_SNP6 [C/G] on CDS site 716. The CBF-A15_SNP4, CBF-A15_SNP5, CBF-A15_SNP6 and CBF-A15_inde1 are located upstream of the identified domains/motifs. All five AA substitutions regions show no positive or negative selection (Fig. 8a, Additional file 13: Figure S8). The CBF-A14 AA haplotypes do not result in AA changes (Additional file 14: Figure S9). The CBF-A18 haplotypes carry 14 AA substitutions. However, none of these is associated with FT or underlies negative or positive selection (Additional file 15: Figure S10).

#### Vernalisation genes (VRN-A1 and VRN-B3)

Nine homologue AA sequences of VRN-A1 and VRN-B3 were identified (Additional file 9: Table S4). The RaptorX analysis of VRN-A1 identified protein structures from MADS-box/MEF2 (myocyte enhancer factor 2) domain (3kov) and MEF2A (1egw), which are located in the same conserved domain. In addition, the keratin-like (K) domain (4ox0) was identified. The complete complex was described by Theissen et al. [102] and Kaufmann et al. [103] with a MADS DNA binding (M) domain, a type II transcription factor containing the Intervening (I) domain, a Keratin-like coiled-coil (K) domain and a C-terminal (C) domain (MIKC-type). The parameters of protein structure modelling are shown in Table 6. The VRN-A1_SNP1 [C/T/Y] on CDS site 349 generated an AA change of Leu/Phe in the K domain between α helix three and α helix four. Furthermore, in the region of this AA substitution, no positive or negative selection was identified (Fig. 8b, Additional file 16: Figure S11).

The association study revealed that *VRN-B3* is significantly associated with FT only in the SNP/indel analysis. The protein prediction analysis identified an Hd3A protein (3axy), which is a mobile flowering signal in rice (Table 6). This flowering time family protein contains four α helices, seven β strands and one segment B [104]. The VRN-B3_SNP1 [C/G] on CDS site 19 generates an AA change of His/Asp directly before the start of the α helix 1. Furthermore, this site is subject to significant negative selection (Fig. 8c, Additional file 17: Figure S12).

#### Photoperiod response genes (PPD-B1 and PPD-D1)

The association study revealed that the gene *PPD-B1* is significantly associated to FT only in the haplotype analysis and *PPD-D1* in both analyses. The NCBI protein BLASTX of the PPD-B1 and PPD-D1 AA haplotype sequences showed nine homologues (Additional file 9: Table S4). On the basis of the protein BLAST analysis, the Pseudo-Receiver domain and the CONSTANS motif of the pseudo-response regulator (PRR) protein family were identified [39, 40]. In the RaptorX analysis the protein models of a putative response regulator domain (3t6k) and response regulator receiver domain (3jte) were identified (Table 6). These domains contain five α helices and five β strands. The PPD-B1 haplotype AA exhibited three AA changes. The PPD-B1_SNP2 [A/G] on CDS site 304 generated an AA change of Arg/Gly and PPD-B1_SNP3 [A/G] on CDS site 368 generated an AA change of Asn/Asp. Both are located in the Pseudo-Receiver domain. The PPD-B1_SNP2 is located in the α helix 3 and PPD-B1_SNP3 between β strand 4 and α helix 4. The third AA change from Asp to Asn is caused by PPD-B1_SNP5 [G/A] on CDS site 623. All three AA substitution sites show no significant positive or negative selection (Fig. 8d, Additional file 18: Figure S13). The associated PPD-D1_indel1 on CDS site 1266 to 1270 bp produced a stop codon on AA position 470. Therefore, haplotype one has no CONSTANS motif. All other AA changes between haplotype two and three did not show significant association in the SNP/indel association study (Additional file 19: Figure S14).

### *In silico* promoter analysis

The significantly associated CBF-A5_inde1 [C/−], which is located 83 bp upstream of the start codon, entails no changes on the promoter regulatory sites and has no influence on the transcription of the *CBF-A5* gene. Also, the associated polymorphic sites CBF-A13_SNP1 of the *CBF-A13* promotor and the sites CBF-A14_indel1, CBF-A14_SNP1, CBF-A14_SNP2 and CBF-A14_SNP3 of the *CBF-A14* promoter show no regulatory site changes which are indicative for a modification of the gene transcription.

## Discussion

After hybridisation, domestication and breeding activities have shaped the genome of bread wheat and reduced the level of genetic diversity which is nowadays the major limiting factor in breeding of cultivars resistant to biotic and abiotic stresses [105]. Therefore, sequencing of candidate genes in large collections is a tool to rediscover hidden genetic diversity and use this in breeding.

### LD and diversity

The genotype group from Asia shows a very small diversity with 25 polymorphic sites out of 81 genotypes. That implies that these genotypes are very close to each other based on the analyzed genes. The polymorphic sites of the different chromosomes show a high LD among themselves but not between the different chromosomes. Consequently, the polymorphic sites which are in a high LD are inherited together. In contrast, genotypes from North America show a very high diversity with 88 polymorphic sites in 38 genotypes. That implies that these genotypes are very distant to each other based on the analyzed genes. The gene cluster of *CBFs* (*CBF-A3*, *CBF-A10*, *CBF-A13*, *CBF-A14* and *CBF-A15*) on chromosome 5A shows a very high LD of r^2^ = 1. Consequently, this gene cluster has not been divided by meiotic events but separated from Cab also located on chromosome 5A. An intermediate diversity was detected in genotypes from Europe with 51 polymorphic sites in 116 genotypes. The LD analysis shows a very high LD within three blocks located on chromosome 5A but not between. Consequently the gene cluster (*CBF-A3*, *CBF-A10*, *CBF-A13*, *CBF-A14* and *CBF-A15*), *Cab* and *VRN-A1* are inherited independently.

### Association study

FT is a highly complex and important trait of winter wheat that is usually studied using QTL and expression profiling approaches [47, 106, 107]. In this study we are presenting, to our best knowledge, the first large scale candidate gene based association analysis of FT in wheat.

Accordingly, we identified significantly associated polymorphisms (SNPs/indels) in eleven studied genes as well as respective haplotypes. Eight of these were detected in both approaches (*CBF-A3*, *CBF-A5*, *CBF-A10*, *CBF-A13*, *CBF-A14*, *CBF-A15*, *PPD-D1* and *VRN-A1*). Out of the seven *CBF* genes, which are members of a large gene family that were investigated in this study, six revealed FT association in the SNP/indel and haplotype method. Associated polymorphisms at the five candidate genes *CBF-A3*, *CBF-A10*, *CBF-A13*, *CBF-A14* and *CBF-A15* are located at the FR-A2 locus [46, 47] on wheat chromosome 5A. The other two *CBF* members, i.e. *CBF-A5* and *CBF-A18,* are located on chromosomes 7A and 6A and do not belong to the FR-A2 locus. Additionally, all the SNPs identified in *CBFs* on chromosome 5A are in a very high LD (r^2^ = 0.92 to 1), indicating localisation at the same chromosomal region without or very rare recombination. This result confirms previous knowledge of strong linkage of *CBF* genes and emphasises the importance of the FR-A2 locus in frost response. On the other hand, results reveal that not all members of the *CBF* family are involved in FT [50, 108]. Based on the occurrence of only two haplotypes of the *CBF* genes and the fact that they are very closely linked, genotypes can be divided into two groups. Group one with 193 genotypes which shows 15% better winter survival in comparison to group two (about 42 genotypes) (Table 4). Therefore, breeding efforts in combining two FT haplotypes are highly desirable towards creating elite cultivars exhibiting high FT.

The CBF-A3_SNP2 [C/G] which is significantly associated with FT (*P* = 6.28 × 10^− 10^) results in Ala/Gly AA change. This AA is localised in the α helix of the AP2 domain and shows a high conservation of Ala in the AA alignment with homologues. Only the AA haplotype two had a Gly which corresponds to a significant reduction in winter survival of 15.01% in the SNP/indel association study. Also haplotype one shows a 15.01% better winter survival compared to haplotype two with Gly in the haplotype association study (Tables 4 and 5). Allen et al. [28] describe that Ala contributes to the stabilisation of the protein structure by its hydrophobic side chain. Through the AA change from Ala to Gly it is possible that the Gly of AA haplotype two loses or impairs the functionality as a transcription factor. Additionally, the dN/dS ratio analysis shows that the CBF-A3_SNP2 site underlies negative selection (Fig. 7), reflecting the association effects. In detail, haplotype two with an Gly on CBF-A3_SNP2 site shows lower winter survival of 15.01% compared to haplotype one.

The SNP/indel association study of *CBF-A5* exon SNPs revealed no significant association. In contrast, the exon haplotype is significantly associated with a reduced winter survival of 19.51% respectively 18.01% of exon haplotype three (Ser on site CBF-A5_SNP2) compared to haplotype one respectively two. Due to the fact that the exon haplotype three consists only of three genotypes it was not identified in the SNP/indel association analysis (Table 5, Additional file 10: Figure S5).

The significantly associated SNPs and indels of *CBF-A13* may disorganise the protein structure very strongly. The one bp CBF-A13_indel1 of haplotype two changed the complete AA sequences from the seventh AA onwards and a stop codon is present after 74 AAs. Since only six AAs are identical to the reference protein sequence, a complete loss of function may be assumed. The 32 bp deletion (CBF-A13_indel2) of haplotype one also generates a frame shift from AA 45 on and a stop codon after 135 AAs. Hence, the AA haplotype two exhibits no similarity and the AA haplotype one shows a very low uSeqId of 15 but an appropriate *P*-value of 4.79 × 10^− 4^ compared to the AP2 domain. Therefore, it is most likely that both proteins are non-functional. However, haplotype one shows about 6.20% higher winter survival in the SNP/indel association study and in the haplotype association study (9.80%) compared to haplotype two. Consequently, it is possible that the proteins of AA haplotype one have an increased transcription factor efficiency compared to the proteins of AA haplotype two. On the other hand the association may be due to the very high LD within the FR-A2 locus.

Regarding the two AA haplotypes of CBF-A15 an AA change in the AP2 domain from Ala to Val was identified which matched to the statistically significant associated CBF-A15_SNP3 [C/T] with a *P*-value of 6.28 × 10^− 10^. Ala is conserved at this position at the nine homologue AA sequences. The haplotype two which comprises Val in the AA sequence shows 15.01% less winter survival in the SNP/indel association study and 15.01% in the haplotype association study. That indicates a functional loss, although Ala as well as Val possess a hydrophobic side chain and are very similar in molecule size. A functional loss due to this AA change is unlikely. On the other hand, no statistically significant positive or negative selection at this site was detected.

Only the whole gene haplotype of *CBF-A18* is significantly associated to FT. Due to the MAF selection, no association study could be performed for all other haplotype components and polymorphic sites of *CBF-A18* (Table 5).

All other SNPs/indels of the seven *CBFs,* which resulted in AA changes, may be involved in FT but they are not located in the highly conserved AP2 domain. But, the CBF-A3_SNP4, which is located upstream of the AP2 domain, shows conserved AA sites. This may play an important regulatory role in FT. The associated polymorphisms of *CBF-A5* and *CBF-A14* are located within the promoter and the in silico promoter analysis of these haplotypes shows no promoter region differences which explain modifications in gene transcription.

This study revealed significantly associated SNPs/indels and haplotypes in seven *CBF* genes. Out of the associated polymorphisms two SNPs were identified, which resulted in an AA change in the highly conserved AP2 domain of the CBF-A3 and CBF-A15 protein. Both *CBF* genes were identified as important FT genes in *Triticum monococcum* and *Triticum aestivum* [47, 59, 61–63]. All this leads to the conclusion that SNP CBF-A3_SNP2 is the most interesting *CBF* allele for FT improvement and at the same time CBF-A15_SNP3 is the second most important one*.* Further details remain to be revealed by investigations in the future such as complementation of promising alleles in spring or winter wheat varieties or protein functionality analysis of these alleles.

Identification of the VRN-A1_SNP1 [C/T/Y] revealed that the genotypes with the base T and the genotypes with the ambiguous nucleotide Y increase FT by 1.35% respectively 0.11% in comparison to the genotypes with the base C. The exon haplotype association study shows stronger effects. In detail, haplotype three (T) and haplotype four (Y) increase FT by 12.44% respectively 11.21%, in comparison to haplotype two (C). Chen et al. [53] and Eagles et al. [55] also identified the C and Y allele and Diaz et al. [109] and Zhu et al. [107] described that the C allele is associated with a lower *VRN-A1* copy number and the Y allele with a higher copy number. In addition, Zhu et al. [107] described that an increase of the *VRN-A1* copy number is associated with improved FT among the FR-A2-T allele of the *CBF12* and *CBF15* genes but not among the FR-A2-S which are reflected in our haplotypes one and two, respectively. However, the VRN-A1_SNP1 generates an AA change on a Leu conserved side in the K-domain of a MIKC-type transcription factor between α helix three and α helix four to a Phe. Puranik et al. [110] showed that this Leu stabilises the kink region between α helix three and α helix four by extensive intra-molecule hydrophobic interactions of multiple Leu residues. Both AA have hydrophobic side chains but Phe with its benzene ring strongly differs from Leu for its steric requirements. Therefore, it is possible that the Phe increases the angle between both α helices due to its bulkiness and the attachment to the target sequence of the transcription factor is improved. Additionally the VRN-A1_SNP1 underlies no negative or positive selection.

The *VRN-B3* gene is significantly associated to FT only in the SNP/indel association study. The polymorphism VRN-B3_SNP1 [C/G] shows a *P*-value of 3.76 × 10^− 2^. This SNP generates a His/Asp AA change. The genotypes with His show a higher winter survival of 3.05% compared to those containing Asp (Table 4). Additionally this site shows a high conservation of Asp regarding all nine homologue AA sequences and underlies significant negative selection. All this data indicates that *VRN-B3* and respective homologous play a role in FT.

The polymorphisms of the *PPD-B1* gene merely show significance in the haplotype association study. The exon haplotype three shows 5.11% better winter survival than haplotype four. The difference between both haplotypes is the PPD-B1_SNP2 [A/G] which generates an Arg/Gly change in the α helix 3 of the Pseudo-Receiver domain. The Arg is highly conserved for grasses [39, 40]. Therefore, it is possible that the Gly from haplotype three has a positive effect on FT. Another AA change in the Pseudo-Receiver domain from Asn to Asp is caused by the PPD-B1_SNP3 [A/G]. Asp at this position is highly conserved. Only haplotype one and the homologue AA of *Triticum aestivum* show Asn resulting in a 9.25% decreased winter survival in comparison to haplotype four. The haplotype two, which is associated with PPD-B1_SNP5 [G/A], and an Asp/Asn AA change shows 28.28% less winter survival in comparison to haplotype four. The most tolerant haplotype four originates from the Asia group. The position of this AA is 51 AAs downstream of the Pseudo-Receiver domain and is slightly conserved. Association of the haplotypes one (three observations) and two (five observations) are based on very few observation and therefore the results should be interpreted with caution. The dN/dS analysis revealed no significant negative or positive selection for all three AA substitutions (PPD-B1_SNP2, PPD-B1_SNP3 and PPD-B1_SNP5) (Additional file 18: Figure S13).

The associated indel of *PPD-D1* has an effect of 3.96% in the SNP/indel association study (Table 4). Haplotype one (with a deletion) shows 3.62% better winter survival compared to haplotype two in the haplotype association study (Table 5). As a result of this deletion, a stop codon on AA position 470 occurs and the CONSTANS motif is missing. The consequence is that the PPD-D1 protein cannot interact with the CO protein and the flowering control pathway is interrupted [38]. Furthermore, the interaction between the flowering time and FT pathway is disturbed (Additional file 19: Figure S14).

**Fig. 9:**
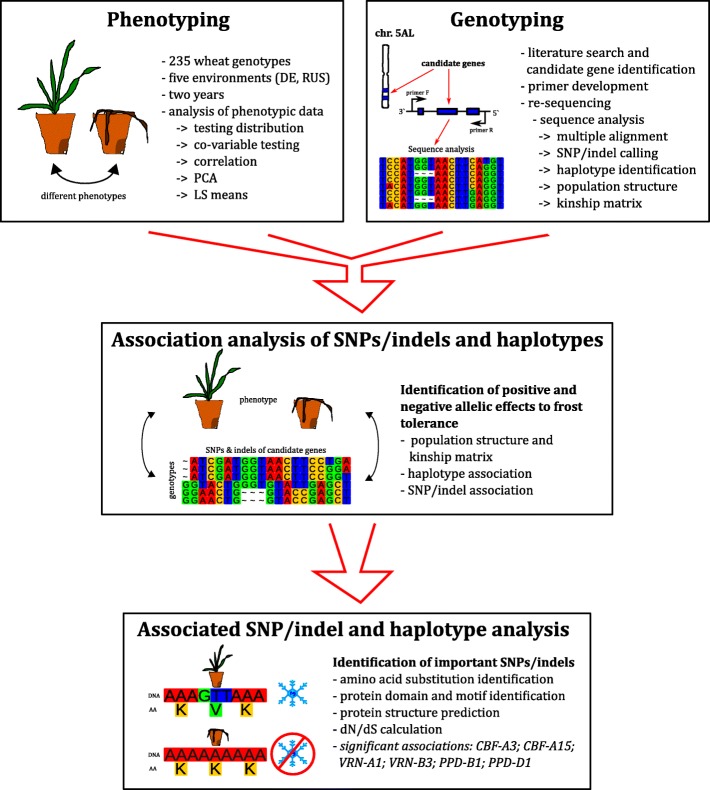
Workflow for identifying associations for frost tolerance in wheat

This study demonstrated polymorphisms significantly associated with FT and the importance of the AA changes of seven *CBF* gene family members and the *VRN-A1*, *VRN-B3*, *PPD-B1* and *PPD-D1* genes (Fig. 9). These results may be used to design highly frost tolerant wheat cultivars via gene engineering or classical breeding. To achieve this, six associated genes (*CBF-A3*, *CBF-A15*, *VRN-A1*, *VRN-B3*, *PPD-B1* and *PPD-D1*) have to be combined, employing the alleles which show the strongest positive effect in FT. We suggest a wheat cultivar with the haplotype one of both *CBF* genes, haplotype three of *PPD-B1*, haplotype one of *PPD-D1*, haplotype three of *VRN-A1* and VRN-B3_SNP1 of *VRN-B3* to create a genotype with the theoretically highest FT regarding the investigated genes. Additional candidate gene based association genetics studies in the field of FT should focus on the *COR* (cold-regulated) genes and proteins.

## Conclusion

In FT research and especially with respect to genome based breeding, it is important to identify genes and alleles involved in cold response. Based on the phenotypic data from German and Russian field trials, this study illustrates the importance of the *CBF* genes of the FR-A2 locus, *VRN-A1*, *VRN-B3*, *PPD-B1* and *PPD-D1* for FT of wheat. Polymorphisms in *CBF-A3*, *CBF-A5*, *CBF-A10*, *CBF-A13*, *CBF-A14*, *CBF-A15*, *CBF-A18*, *VRN-A1*, *VRN-B3*, *PPD-B1* and *PPD-D1* were identified which are significantly associated with FT. Besides this, we detected significantly associated polymorphisms and consequential AA substitutions in important cold response protein domains. The results of this study demonstrated that the candidate based association genetics approach is a very useful method to identify alleles positively contributing to frost tolerance in hexaploid wheat. The FT associated SNPs/indels and haplotypes identified may be used for developing diagnostic markers for marker assisted selection (MAS) for frost tolerance in wheat.

## Additional files

Additional file 1: **Table S1.** Plant material, origin, LS means of winter survival [%] and winter survival [%] of respective environments. (XLSX 34 kb)

Additional file 2: **Table S2.** PCR and sequencing primers. ^1^Shcherban et al., 2012; ^2^Miller et al., 2006; ^3^Vagujfalvi et al., 2005;^4^Yan et al., 2004; ^5^Beales et al., 2007; ^6^Keilwagen et al., 2014; ^7^Knox et al., 2008; ^8^Babben et al., 2015; ^9^Seki et al., 2011; ^10^Li et al., 2011b (XLSX 19 kb)

Additional file 3: **Data S1.** Java script used to extract differences from a multiple sequence alignment (MSA). (TXT 5 kb)

Additional file 4: **Figure S1.** Scatter plot of mean winter survival and number of days under − 15 °C from ten environments. (PDF 24 kb)

Additional file 5: **Figure S2.** PCA plot of mean winter survival and number of days under − 15 °C from ten environments. (PDF 52 kb)

Additional file 6: **Figure S3.** Localization of 19 candidate genes and corresponding primers in the Chinese Spring Reference assembly v1.0. Next to the gene names, the chromosome number and physical position of the Chinese Spring reference assembly v1.0 are shown in brackets. For each gene the structure is illustrated below. The black lines indicate the genomic sequences, the gray boxes the exons, the black arrows rightward the forward primers and the black arrows leftwards the reverse primers. (PDF 23 kb)

Additional file 7: **Figure S4.** Principal coordinate analysis of 235 wheat cultivars. Three sub-populations based on geographical origin were shown in three colors. Blue, red and green indicate the cultivars from Europe, North America and Asia, respectively. The black dots indicatethe mixture gemplasm from three sub-populations. (PDF 42 kb)

Additional file 8: **Table S3.** Polymorphic sites and haplotypes of association study and their PH-values and FT effects. (XLSX 14 kb)

Additional file 9: **Table S4.** Identities and e values of FT associated AA sequences and homologous AA sequences. (XLSX 21 kb)

Additional file 10: **Figure S5.** Amino acid alignment and nucleotide divergence rates (dN/dS) of *CBF-A5* and nine homologous amino acid sequences. Shownare alignments of two haplotype AA sequences of CBF-A5 and nine homologous plant AA sequences. The numbers above the alignment indicate the sites of AAs. The black line above the alignment illustrates the PKK/RPAGRxKFxETRHP and DSAWR motif, the red line the AP2 domain, the black arrows the β-strands and the black spiral the α-helix. The description of red arrows, black line und red dots is according to Fig. 6. (PDF 54 kb)

Additional file 11: **Figure S6.** Amino acid alignment and nucleotide divergence rates (dN/dS) of *CBF-A10* gene and nine homologous amino acid sequences. Alignments of two haplotype AA sequences of CBF-A10 and nine homologous plant AA sequences. The numbers above the alignment indicate the sites of AAs. The black line above the alignment illustrates the PKK/RPAGRxKFxETRHP and DSAWR motif, the red line the AP2 domain, the black arrows the β-strands and the black spiral the α-helix. The description of red arrow, black line and red dots is according to Fig. 6. (PDF 53 kb)

Additional file 12: **Figure S7.** Amino acid alignment and nucleotide divergence rates (dN/dS) of *CBF-A13* gene and nine homologous amino acid sequences. Illustrated are alignments of two haplotype AA sequences of CBF-A13 and nine homologous plant AA sequences. The numbers above the alignment illustrate the sites of AAs. The black line above the alignment illustrates the PKK/RPAGRxKFxETRHP and DSAWR motif, the red line the AP2 domain, the black arrows the β-strands and the black spiral the α-helix. The description of black line and red dots is according to Fig. 6. (PDF 53 kb)

Additional file 13: **Figure S8.** Amino acid alignment and nucleotide divergence rates (dN/dS) of *CBF-A15* gene and nine homologous amino acid sequences. Illustrated are alignments of two haplotype AA sequences of CBF-A15 and nine homologous plant AA sequences. The numbers above the alignment illustrate the sites of AAs. The black line above the alignment illustrates the PKK/RPAGRxKFxETRHP and DSAWR motif, the red line the AP2 domain, the black arrows the β-strands and the black spiral the α-helix. The description of red arrows, black line and red dots is according to Fig. 6. (PDF 52 kb)

Additional file 14: **Figure S9.** Amino acid alignment and nucleotide divergence rates (dN/dS) of *CBF-A14* gene and nine homologous amino acid sequences. Illustrated are alignments of two haplotype AA sequences of CBF-A14 and nine homologous plant AA sequences. The numbers above the alignment illustrate the sites of AAs. The black line above the alignment illustrates the PKK/RPAGRxKFxETRHP and DSAWR motif, the red line the AP2 domain, the black arrows the β-strands and the black spiral the α-helix. The description of black line and red dots is according to Fig. 6. (PDF 51 kb)

Additional file 15: **Figure S10.** Amino acid alignment and nucleotide divergence rates (dN/dS) of *CBF-A18* gene and nine homologous amino acid sequences. Illustrated are alignments of two haplotype AA sequences of CBF-A18 and nine homologous plant AA sequences. The numbers above the alignment illustrate the sites of AAs. The black line above the alignment illustrates the PKK/RPAGRxKFxETRHP and DSAWR motif, the red line the AP2 domain, the black arrows the β-strands and the black spiral the α-helix. The description of black line und red dots is according to Fig. 6. (PDF 54 kb)

Additional file 16: **Figure S11.** Amino acid alignment and nucleotide divergence rates (dN/dS) of *VRN-A1* gene and nine homologous amino acid sequences. Illustrated are alignments of three haplotype AA sequences of VRN-A1 nine homologous plant AA sequences. The numbers above the alignment illustrate the sites of AAs. The black line above the alignment illustrates the I domain and C domain, the red line the MADS box domain and K domain, the black arrows the β-strands and the spirals the α-helices. The description of red arrow, black line and red dots is according to Fig. 6. (PDF 79 kb)

Additional file 17: **Figure S12.** Amino acid alignment and nucleotide divergence rates (dN/dS) of *VRN-B3* gene and nine homologous amino acid sequences. Illustrated are alignments of three haplotype AA sequences of VRN-B3 nine homologous plant AA sequences. The numbers above the alignment illustrate the sites of AAs. The black line above the alignment illustrates the segment B, the red line the flowering time (FT)-family protein, the black arrows the β-strands and the spirals the α-helices. The description of red arrow, black line and red dots is according to Fig. 6. (PDF 51 kb)

Additional file 18: **Figure S13.** Amino acid alignment and nucleotide divergence rates (dN/dS) of *PPD-B1* gene and nine homologous amino acid sequences. Illustrated are alignments of four haplotype AA sequences of PPD-B1 and nine homologous plant AA sequences. The numbers above the alignment illustrate the sites of AAs. The red line above the alignment illustrates the Pseuodo Receiver domain and COSTANS motif. The description of red arrows, black line and red dots is according to Fig. 6. (PDF 8583 kb)

Additional file 19: **Figure S14.** AA alignment and nucleotide divergence rates (dN/dS) of *PPD-D1* gene and nine homologous amino acid sequences. Illustrated are alignments of four haplotype AA sequences of PPD-D1 and nine homologous plant AA sequences. The numbers above the alignment illustrate the sites of AAs. The red line above the alignment illustrates the Pseudo Receiver domain and COSTANS motif. The description of black line and red dots is according to Fig. 6. (PDF 7539 kb)

## Abbreviations

AA: Amino acid
ANCOVA: Analysis of covariance
AP2/EREBP: APETALA2/Ethylene response element binding protein
BLAST: Basic local alignment search tool
CAB: Calcium-binding EF-hand family protein-like
CBF: C-repeat binding factor
CDS: Coding DNA sequence
CO: CONSTANS
COR/LEA: Cold-responsive/late embryogenesis–abundant
CRT/DRE: C-repeat/dehydration-responsive element
CV: Coefficient of variation
DEM: Defective embryo and meristems
DH: Doubled haploid
DN/DS: Nucleotid divergence rate
FR: Frost resistance
FT: Frost tolerance
GBP: Giga-base pairs
GLM: General linear model
GWAS: Genome-wide association study
HD: Haplotype diversity
ICE: Inducer of CBF expression
IFVCNS: Institute of Field and Vegetable Crops
INDEL: Insertion-deletion
IWGSC: International wheat genome sequencing consortium
KBP: Kilo-base pairs
LD: Linkage disequilibrium
LS: Least-Squares
M: Million
MAF: Minor allele frequency
MAFFT: Multiple Alignment tool Fast Fourier Transform
MCMC: Markov Chain Monte Carlo
MEF: Myocyte enhancer factor
MLM: Mixed linear model
MSA: Multiple sequence alignment
NGS: Next generation sequencing
NT: Nulli-tetrasomic
PCA: Principal component analysis
PCOA: Principal coordinate analysis
PPD: Photoperiod
PRR: Pseudo-response regulator
QTL: Quantitative trait locus
RV: Reaction volume
SNP: Single nucleotide polymorphism
T: Tonne
TACR7: *Triticum aestivum* cold-regulated 7
URGI: Unité de Recherche Génomique Info
UTR: Untranslated region K: number of populations
VRN: Vernalisation
