# Supplementary figures and images for: Association genetics studies on frost tolerance in wheat (Triticum aestivum L.) reveal new highly conserved amino acid substitutions in CBF-A3, CBF-A15, VRN3 and PPD1 genes
Source: BMC Genomics. 2018 May 29;19:409. doi: 10.1186/s12864-018-4795-6 (PMC5975666; doi:10.1186/s12864-018-4795-6)

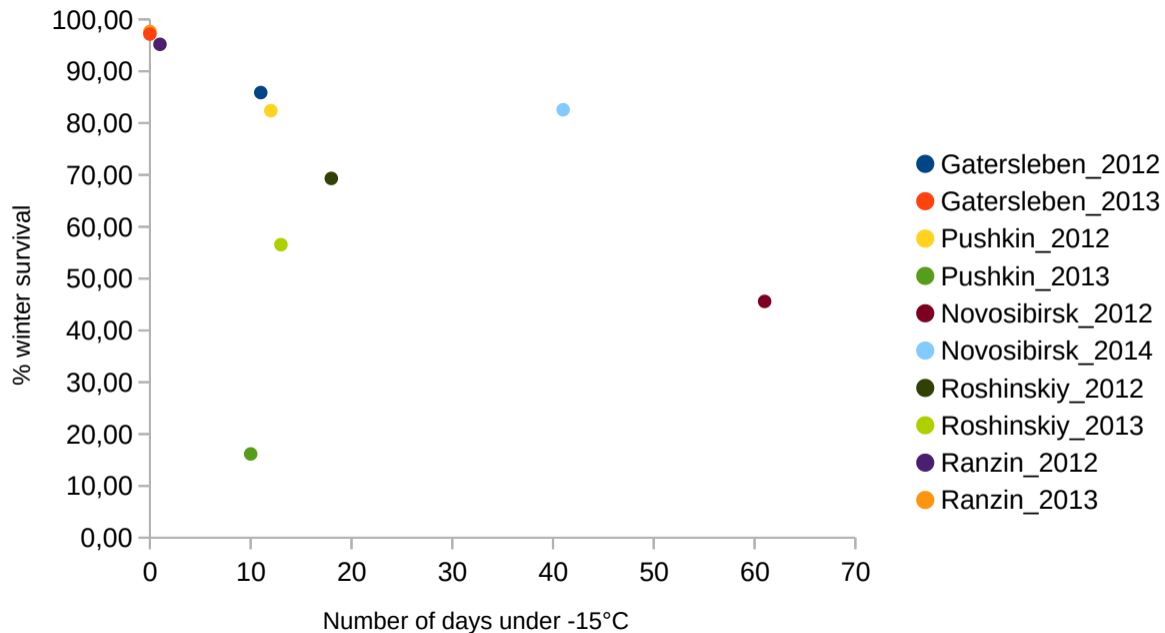

Supplement: Supplementary file 4 — Figure S1. Scatter plot of mean winter survival and number of days under − 15 °C from ten environments. (PDF 24 kb) [file 12864_2018_4795_MOESM4_ESM.pdf]

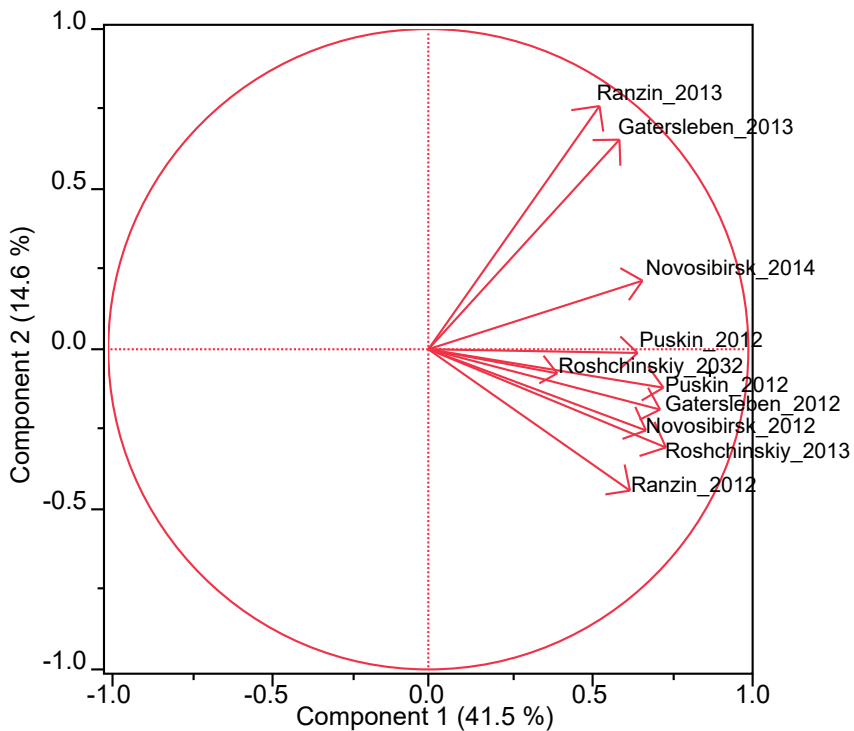

Supplement: Supplementary file 5 — Figure S2. PCA plot of mean winter survival and number of days under − 15 °C from ten environments. (PDF 52 kb) [file 12864_2018_4795_MOESM5_ESM.pdf]

Factorial analysis: (Axes 1 / 2)

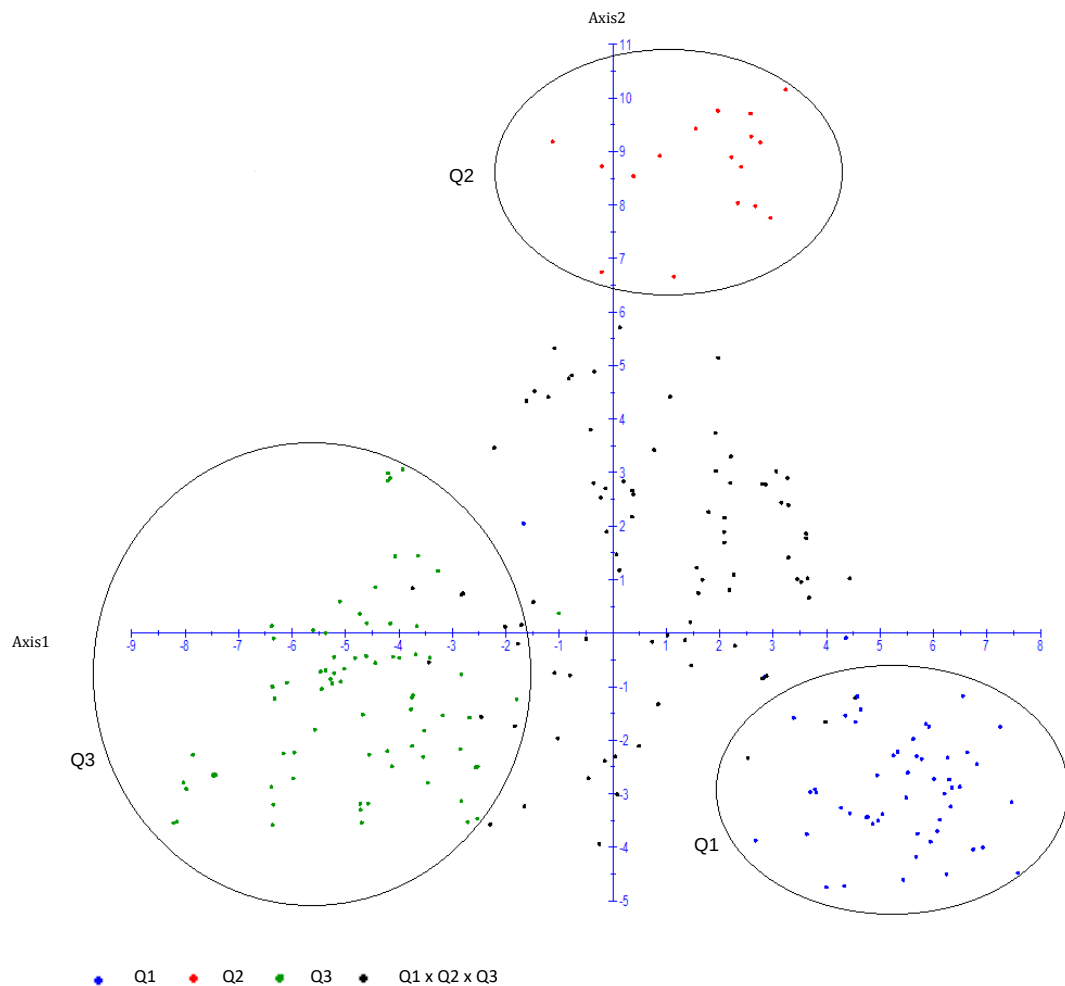

Supplement: Supplementary file 7 — Figure S4. Principal coordinate analysis of 235 wheat cultivars. Three sub-populations based on geographical origin were shown in three colors. Blue, red and green indicate the cultivars from Europe, North America and Asia, respectively. The black dots indicatethe mixture gemplasm from three sub-populations. (PDF 42 kb) [file 12864_2018_4795_MOESM7_ESM.pdf]

# CBF-A5

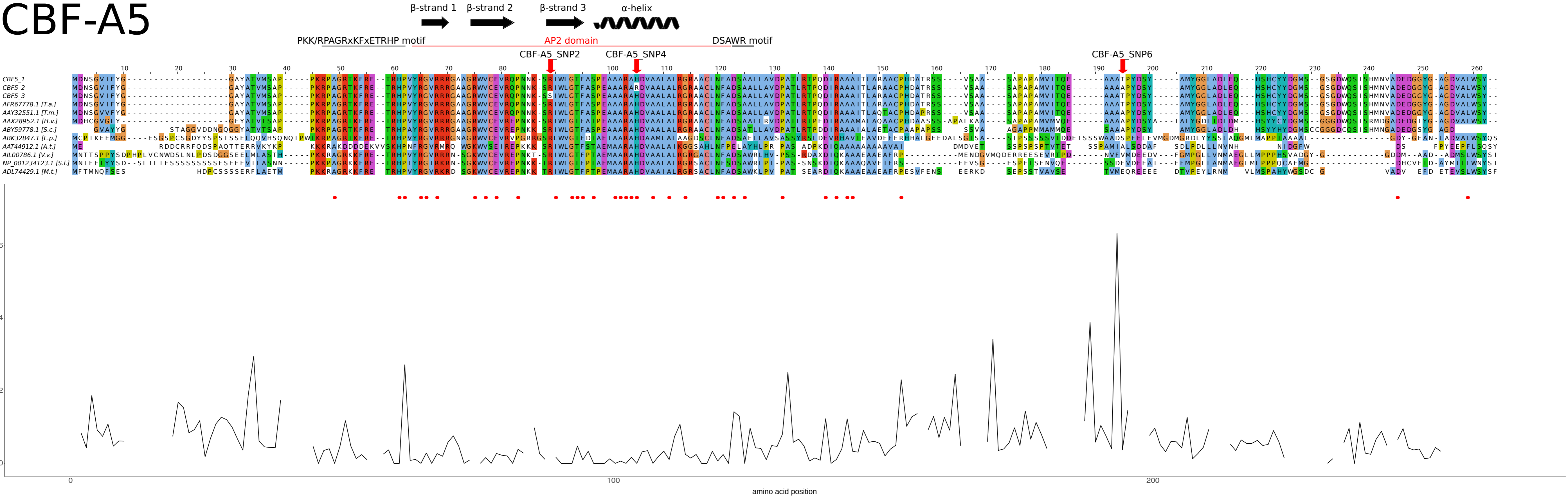

Supplement: Supplementary file 10 — Figure S5. Amino acid alignment and nucleotide divergence rates (dN/dS) of CBF-A5 and nine homologous amino acid sequences. Shownare alignments of two haplotype AA sequences of CBF-A5 and nine homologous plant AA sequences. The numbers above the alignment indicate the sites of AAs. The black line above the alignment illustrates the PKK/RPAGRxKFxETRHP and DSAWR motif, the red line the AP2 domain, the black arrows the β-strands and the black spiral the α-helix. The description of red arrows, black line und red dots is according to Fig. 6. (PDF 54 kb) [file 12864_2018_4795_MOESM10_ESM.pdf]

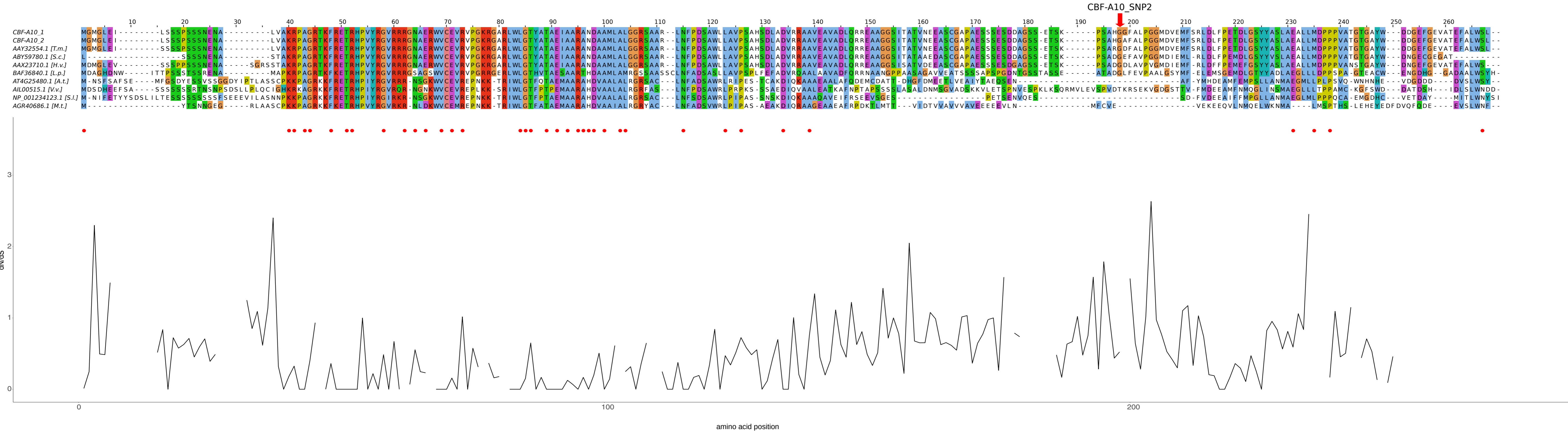

Supplement: Supplementary file 11 — Figure S6. Amino acid alignment and nucleotide divergence rates (dN/dS) of CBF-A10 gene and nine homologous amino acid sequences. Alignments of two haplotype AA sequences of CBF-A10 and nine homologous plant AA sequences. The numbers above the alignment indicate the sites of AAs. The black line above the alignment illustrates the PKK/RPAGRxKFxETRHP and DSAWR motif, the red line the AP2 domain, the black arrows the β-strands and the black spiral the α-helix. The description of red arrow, black line and red dots is according to Fig. 6. (PDF 53 kb) [file 12864_2018_4795_MOESM11_ESM.pdf]

# CBF-A13

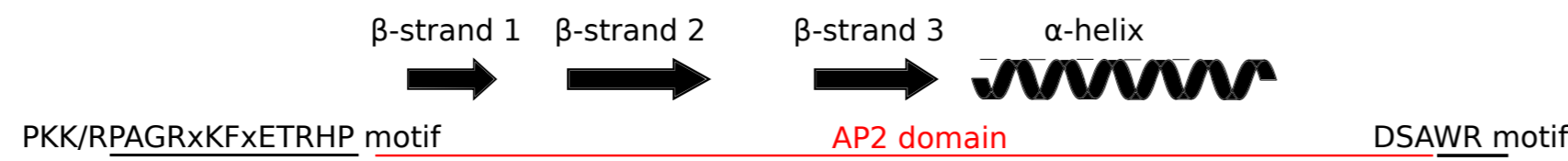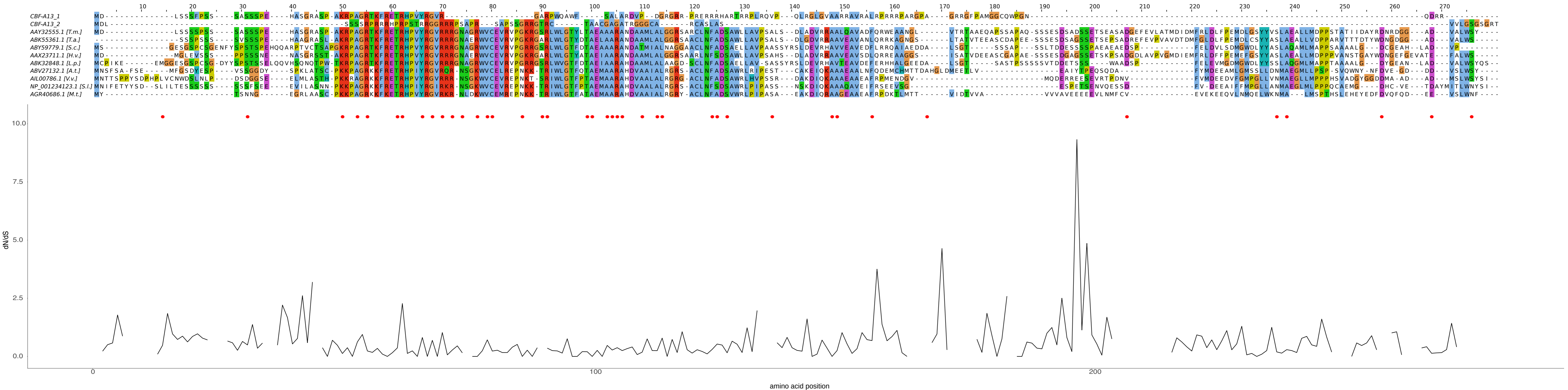

Supplement: Supplementary file 12 — Figure S7. Amino acid alignment and nucleotide divergence rates (dN/dS) of CBF-A13 gene and nine homologous amino acid sequences. Illustrated are alignments of two haplotype AA sequences of CBF-A13 and nine homologous plant AA sequences. The numbers above the alignment illustrate the sites of AAs. The black line above the alignment illustrates the PKK/RPAGRxKFxETRHP and DSAWR motif, the red line the AP2 domain, the black arrows the β-strands and the black spiral the α-helix. The description of black line and red dots is according to Fig. 6. (PDF 53 kb) [file 12864_2018_4795_MOESM12_ESM.pdf]

# CBF-A15

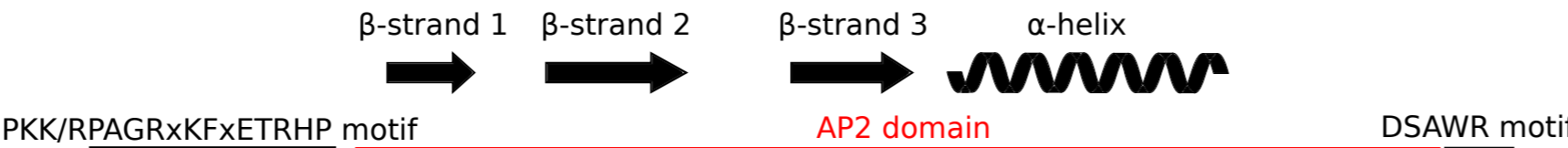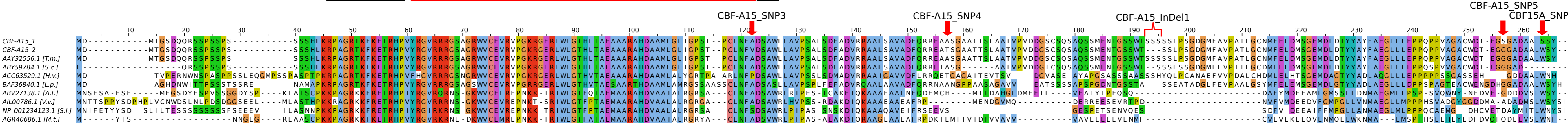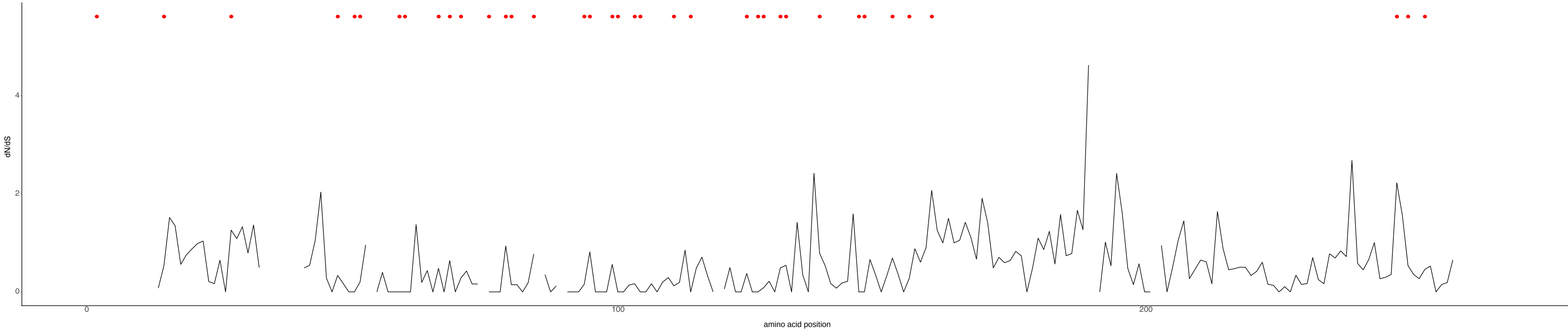

Supplement: Supplementary file 13 — Figure S8. Amino acid alignment and nucleotide divergence rates (dN/dS) of CBF-A15 gene and nine homologous amino acid sequences. Illustrated are alignments of two haplotype AA sequences of CBF-A15 and nine homologous plant AA sequences. The numbers above the alignment illustrate the sites of AAs. The black line above the alignment illustrates the PKK/RPAGRxKFxETRHP and DSAWR motif, the red line the AP2 domain, the black arrows the β-strands and the black spiral the α-helix. The description of red arrows, black line and red dots is according to Fig. 6. (PDF 52 kb) [file 12864_2018_4795_MOESM13_ESM.pdf]

β-strand 1    β-strand 2    β-strand 3    α-helix

PKK/RPAGRxKFxETRHP motif    AP2 domain    DSAWR motif

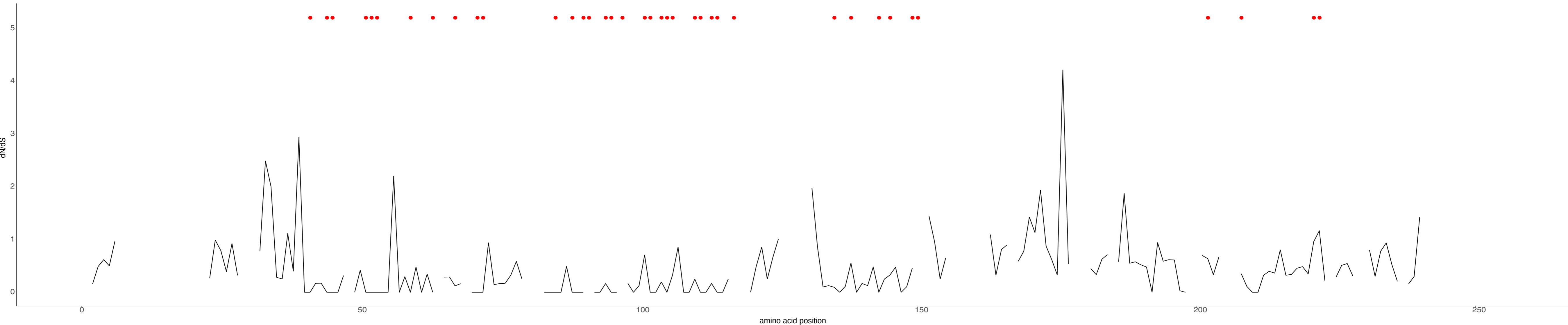

Supplement: Supplementary file 14 — Figure S9. Amino acid alignment and nucleotide divergence rates (dN/dS) of CBF-A14 gene and nine homologous amino acid sequences. Illustrated are alignments of two haplotype AA sequences of CBF-A14 and nine homologous plant AA sequences. The numbers above the alignment illustrate the sites of AAs. The black line above the alignment illustrates the PKK/RPAGRxKFxETRHP and DSAWR motif, the red line the AP2 domain, the black arrows the β-strands and the black spiral the α-helix. The description of black line and red dots is according to Fig. 6. (PDF 51 kb) [file 12864_2018_4795_MOESM14_ESM.pdf]

# CBF-A18

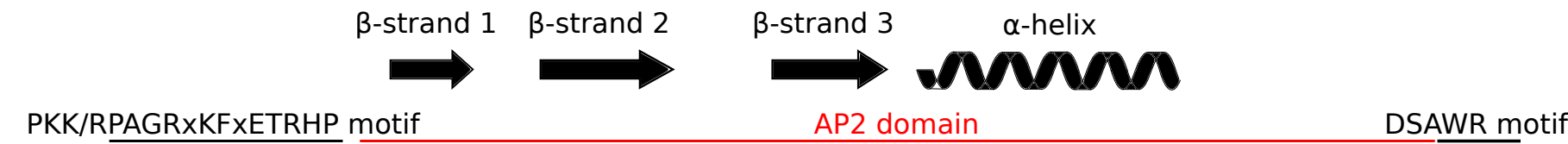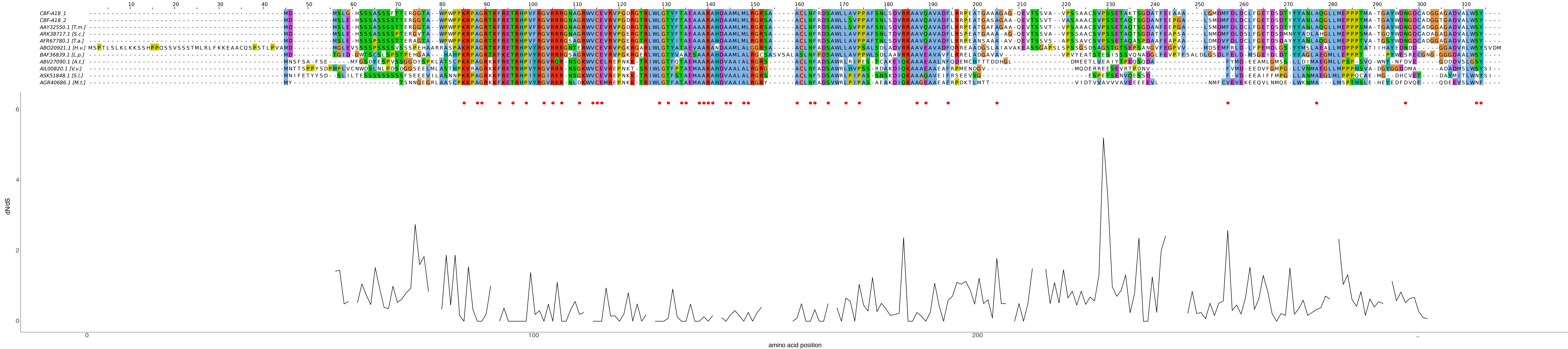

Supplement: Supplementary file 15 — Figure S10. Amino acid alignment and nucleotide divergence rates (dN/dS) of CBF-A18 gene and nine homologous amino acid sequences. Illustrated are alignments of two haplotype AA sequences of CBF-A18 and nine homologous plant AA sequences. The numbers above the alignment illustrate the sites of AAs. The black line above the alignment illustrates the PKK/RPAGRxKFxETRHP and DSAWR motif, the red line the AP2 domain, the black arrows the β-strands and the black spiral the α-helix. The description of black line und red dots is according to Fig. 6. (PDF 54 kb) [file 12864_2018_4795_MOESM15_ESM.pdf]

# VRN-A1

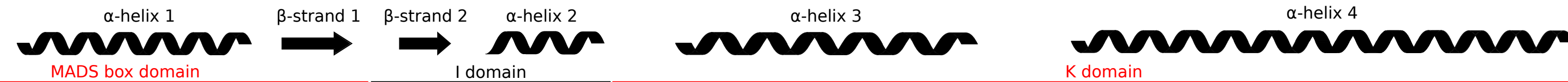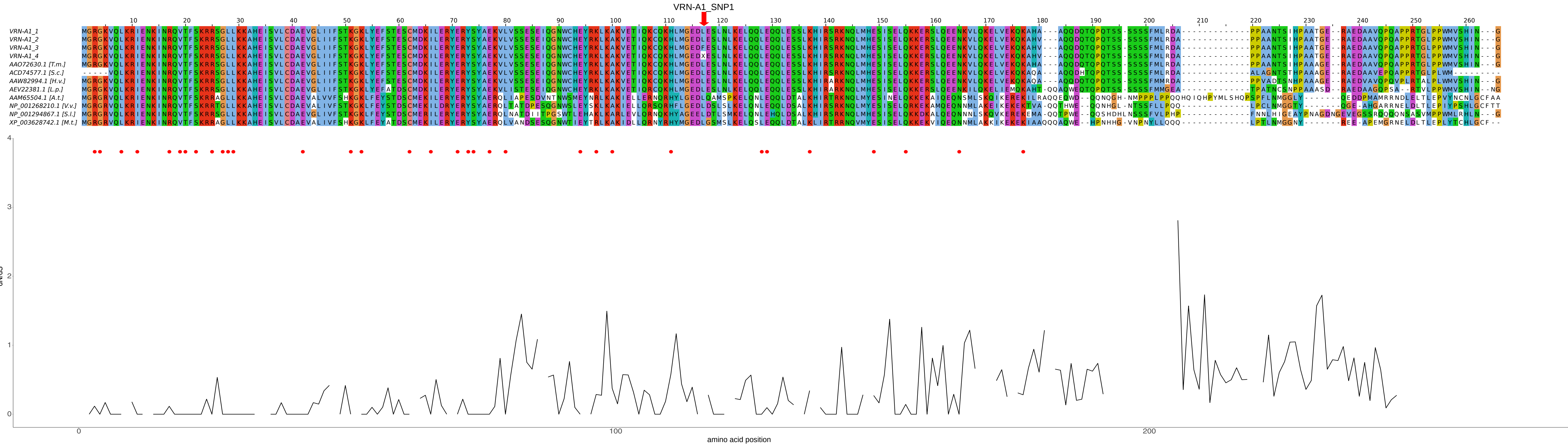

Supplement: Supplementary file 16 — Figure S11. Amino acid alignment and nucleotide divergence rates (dN/dS) of VRN-A1 gene and nine homologous amino acid sequences. Illustrated are alignments of three haplotype AA sequences of VRN-A1 nine homologous plant AA sequences. The numbers above the alignment illustrate the sites of AAs. The black line above the alignment illustrates the I domain and C domain, the red line the MADS box domain and K domain, the black arrows the β-strands and the spirals the α-helices. The description of red arrow, black line and red dots is according to Fig. 6. (PDF 79 kb) [file 12864_2018_4795_MOESM16_ESM.pdf]

# VRN-B3

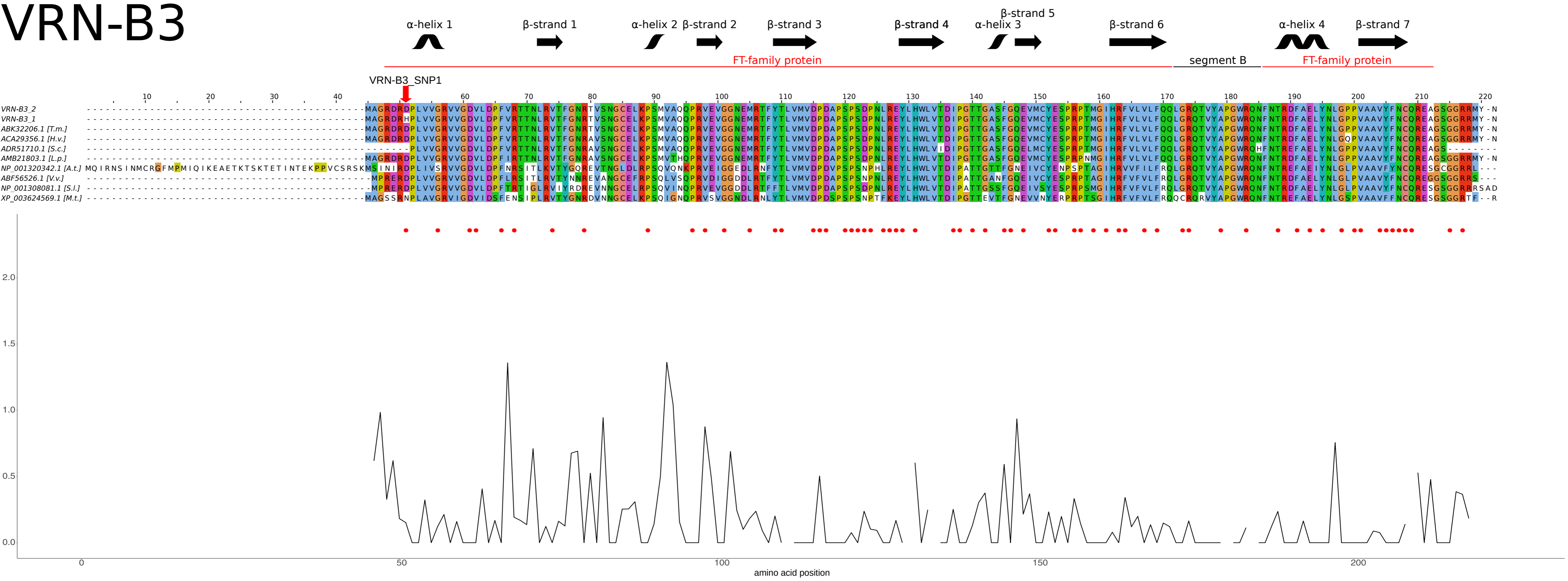

Supplement: Supplementary file 17 — Figure S12. Amino acid alignment and nucleotide divergence rates (dN/dS) of VRN-B3 gene and nine homologous amino acid sequences. Illustrated are alignments of three haplotype AA sequences of VRN-B3 nine homologous plant AA sequences. The numbers above the alignment illustrate the sites of AAs. The black line above the alignment illustrates the segment B, the red line the flowering time (FT)-family protein, the black arrows the β-strands and the spirals the α-helices. The description of red arrow, black line and red dots is according to Fig. 6. (PDF 51 kb) [file 12864_2018_4795_MOESM17_ESM.pdf]

PPD-B1

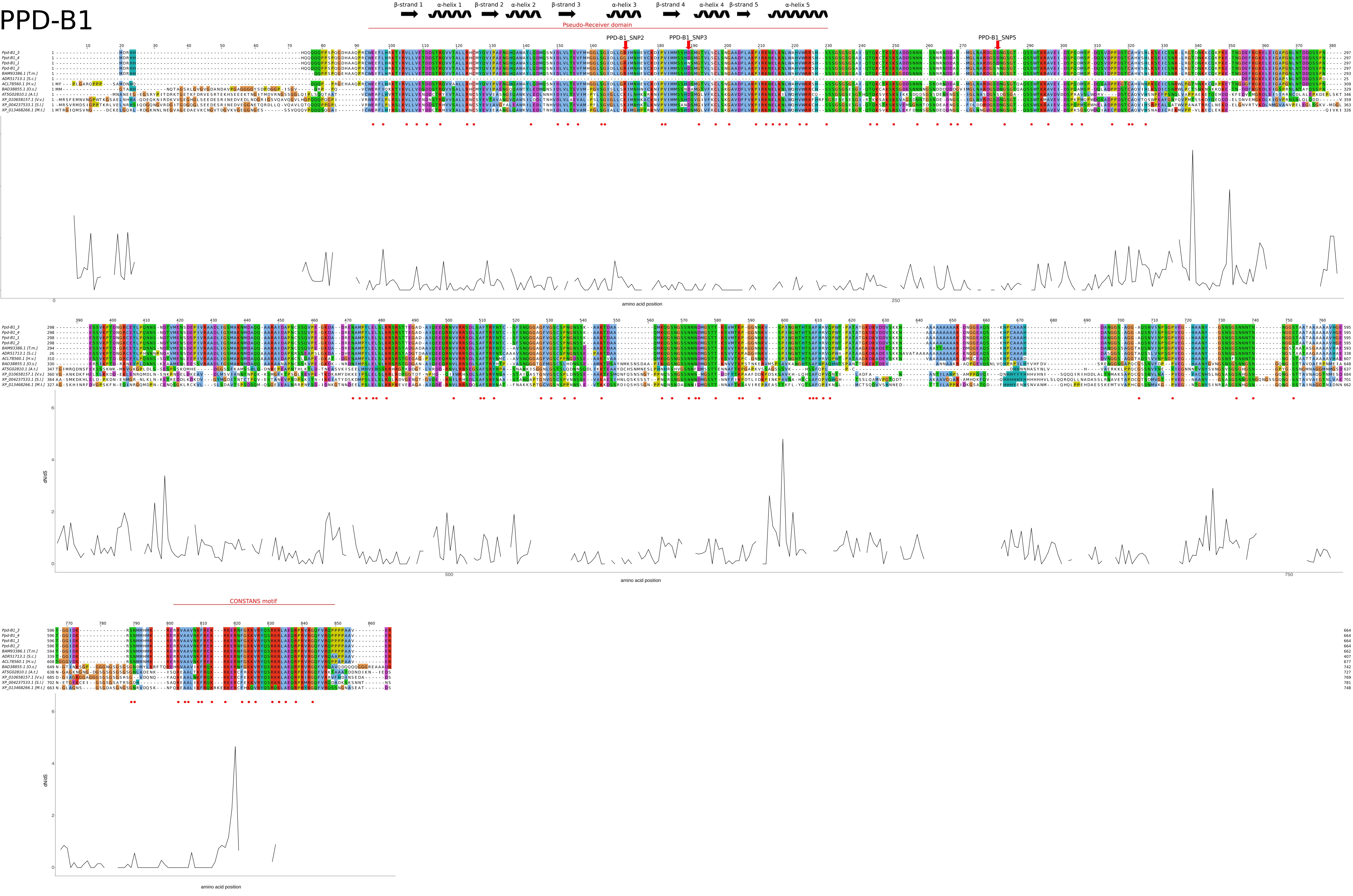

Supplement: Supplementary file 18 — Figure S13. Amino acid alignment and nucleotide divergence rates (dN/dS) of PPD-B1 gene and nine homologous amino acid sequences. Illustrated are alignments of four haplotype AA sequences of PPD-B1 and nine homologous plant AA sequences. The numbers above the alignment illustrate the sites of AAs. The red line above the alignment illustrates the Pseuodo Receiver domain and COSTANS motif. The description of red arrows, black line and red dots is according to Fig. 6. (PDF 8583 kb) [file 12864_2018_4795_MOESM18_ESM.pdf]
