# Supplementary material for: Association genetics studies on frost tolerance in wheat (Triticum aestivum L.) reveal new highly conserved amino acid substitutions in CBF-A3, CBF-A15, VRN3 and PPD1 genes
Source: BMC Genomics. 2018 May 29;19:409. doi: 10.1186/s12864-018-4795-6 (PMC5975666; doi:10.1186/s12864-018-4795-6)
Supplement: Supplementary file 6 — Figure S3. Localization of 19 candidate genes and corresponding primers in the Chinese Spring Reference assembly v1.0. Next to the gene names, the chromosome number and physical position of the Chinese Spring reference assembly v1.0 are shown in brackets. For each gene the structure is illustrated below. The black lines indicate the genomic sequences, the gray boxes the exons, the black arrows rightward the forward primers and the black arrows leftwards the reverse primers. (PDF 23 kb) [file 12864_2018_4795_MOESM6_ESM.pdf]

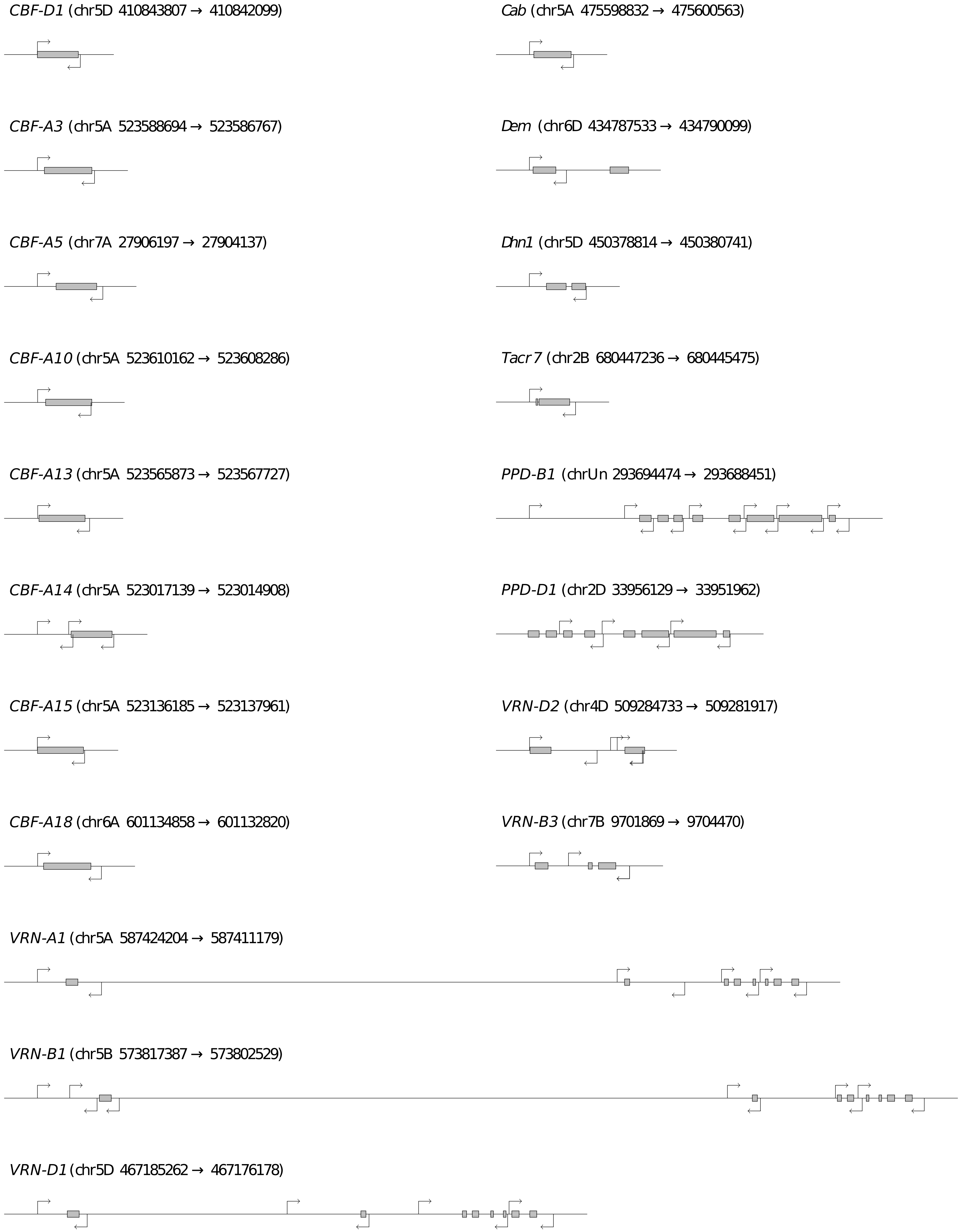
CBF-D1 (chr5D 410843807 → 410842099)
CBF-A3 (chr5A 523588694 → 523586767)
CBF-A5 (chr7A 27906197 → 27904137)
CBF-A10 (chr5A 523610162 → 523608286)
CBF-A13 (chr5A 523565873 → 523567727)
CBF-A14 (chr5A 523017139 → 523014908)
CBF-A15 (chr5A 523136185 → 523137961)
CBF-A18 (chr6A 601134858 → 601132820)
Cab (chr5A 475598832 → 475600563)
Dem (chr6D 434787533 → 434790099)
Dhn1 (chr5D 450378814 → 450380741)
Tacr7 (chr2B 680447236 → 680445475)
PPD-B1 (chrUn 293694474 → 293688451)
PPD-D1 (chr2D 33956129 → 33951962)
VRN-D2 (chr4D 509284733 → 509281917)
VRN-B3 (chr7B 9701869 → 9704470)
VRN-A1 (chr5A 587424204 → 587411179)
VRN-B1 (chr5B 573817387 → 573802529)
VRN-D1 (chr5D 467185262 → 467176178)
