# Supplementary material for: Association genetics studies on frost tolerance in wheat (Triticum aestivum L.) reveal new highly conserved amino acid substitutions in CBF-A3, CBF-A15, VRN3 and PPD1 genes
Source: BMC Genomics. 2018 May 29;19:409. doi: 10.1186/s12864-018-4795-6 (PMC5975666; doi:10.1186/s12864-018-4795-6)
Supplement: Supplementary file 19 — Figure S14. AA alignment and nucleotide divergence rates (dN/dS) of PPD-D1 gene and nine homologous amino acid sequences. Illustrated are alignments of four haplotype AA sequences of PPD-D1 and nine homologous plant AA sequences. The numbers above the alignment illustrate the sites of AAs. The red line above the alignment illustrates the Pseudo Receiver domain and COSTANS motif. The description of black line and red dots is according to Fig. 6. (PDF 7539 kb) [file 12864_2018_4795_MOESM19_ESM.pdf]

PPD-D1

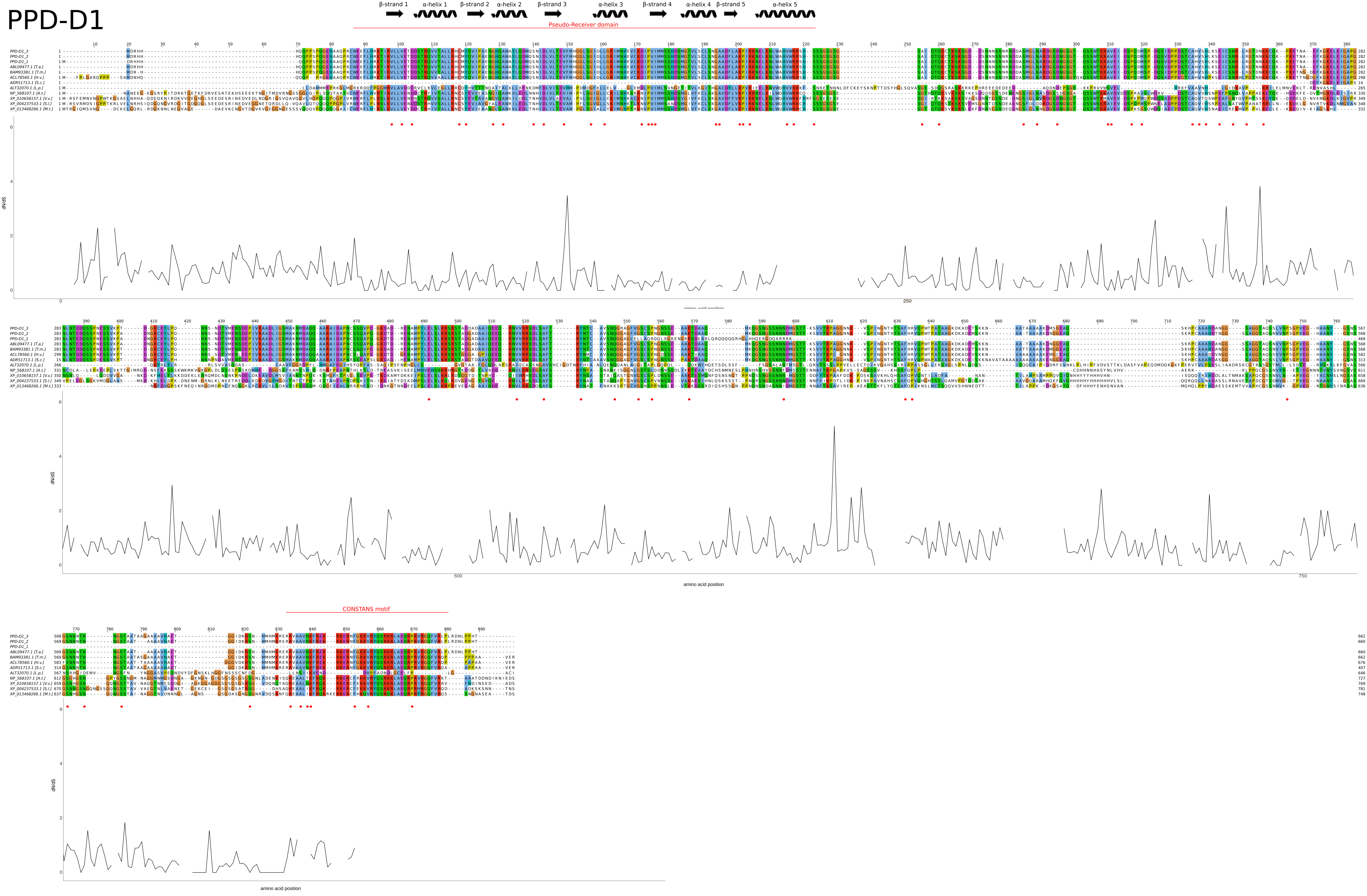

β-strand 1
α-helix 1
β-strand 2
α-helix 2
β-strand 3
α-helix 3
β-strand 4
α-helix 4
β-strand 5
α-helix 5
Pseudo-Receiver domain
PPD-D1_3
PPD-D1_2
PPD-D1_1
ABL09477.1 [T.a.]
BAM93381.1 [T.m.]
ACL78560.1 [H.v.]
ADR51713.1 [S.c.]
ALT32070.1 [L.p.]
NP_568107.1 [A.t.]
XP_010658157.1 [V.v.]
XP_004237533.1 [S.l.]
XP_013468266.1 [M.t.]
dN/dS
amino acid position
CONSTANS motif
